# An RCT META analysis based on the efficacy of Tai Chi exercise therapy on blood pressure and blood lipids in patients with essential hypertension

**DOI:** 10.3389/fcvm.2025.1506912

**Published:** 2025-08-12

**Authors:** Jianan Xu, Yuerong Huang, Lujia Li, Jianwei Zhang, Cuihan Li, Mingyu Liu, Yuxin Ma, Junming Du, Shaojun Lyu

**Affiliations:** ^1^ College of Physical Education and Sport, Beijing Normal University, Beijing, China; ^2^ Department of PE, Peking University, Beijing, China

**Keywords:** Tai Chi, blood pressure, meta-analysis, exercise, public health, hypertension

## Abstract

**Objective:**

Tai Chi is a form of physical and mental exercise. Hypertension, characterized by persistently elevated blood pressure, is a prevalent cardiovascular condition. At present, the effect of Tai Chi exercise cycle on improving the prognosis of patients with essential hypertension, as well as its impact on patients at different stages of hypertension, has not yet been demonstrated. Through meta-analysis, this study systematically evaluated the effects of Tai Chi on hypertensive patients, as well as the roles played by different practice duration, so as to provide evidence-based guidance for future clinical application.

**Methods:**

This meta-analysis, registered in the International Prospective Register of Systematic Reviews (PROSPERO) (CRD42024538168), reviewed RCTs involving Tai Chi interventions for hypertension. Studies were sourced from PubMed, the Cochrane Library, CBM, CNKI, WANFANG, and Embase. Mean differences (MD) and 95% confidence intervals (CI) were calculated using a random-effects model. The sources of heterogeneity were explored using the elimination method one by one and subgroup analysis, supplemented by sensitivity analysis and publication bias assessment.

**Results:**

Seventeen studies were included in the analysis. No adverse events were reported in all the included studies. Tai Chi significantly reduced Systolic Blood Pressure (SBP) (WMD = −9.12; 95% CI = −12.35 to −5.90), Diastolic Blood Pressure (DBP) (WMD = −5.43; 95% CI = −7.22 to −3.64), Total Cholesterol (TC) (WMD = −0.37; 95% CI = −0.62 to −0.12), Triglycerides (TG) (WMD = −0.53; 95% CI = −0.89 to −0.16), and Low-Density Lipoprotein (LDL) levels (WMD = −0.70; 95% CI = −1.12 to −0.28) compared to control groups. Waist circumference (WC) also improved. Subgroup analyses showed that short-term Tai Chi (3 months) was most effective for improving essential hypertension, with a greater impact in grade I hypertension patients compared to those with grade II. No obvious publication bias was found through funnel plots and Egger's test.

**Conclusion:**

The results demonstrated that Tai Chi effectively reduced SBP, DBP, TC, TG, and LDL levels, with the most significant improvements observed at 12 weeks. These findings support the use of Tai Chi as a scientifically validated exercise rehabilitation tool in clinical settings and suggest avenues for further research.

**Systematic Review Registration:**

identifier, PROSPERO [CRD42024538168].

## 1 Introduction

Cardiovascular diseases (CVDs) pose a significant threat to global health, driven by numerous risk factors contributing to their increasing prevalence (1, 2). Recognized traditional risk factors for cardiovascular disease include obesity, hypertension, dyslipidemia, hyperglycemia, poor diet, family history of cardiovascular disease, physical inactivity, and smoking (3, 4). Hypertension affects approximately 275 million individuals in China, with 95% of whom suffer from essential hypertension (EH), making it a major CVD risk factor (5, 6). Left untreated or poorly managed, accelerates EH progression and increases mortality risk.

Lack of physical activity is a major behavioral risk factor for cardiovascular diseases and is often associated with elevated blood pressure, blood lipids, blood sugar, and overweight or obesity (7). Guidelines for cardiovascular disease prevention state that lifestyle changes (including smoking cessation, physical activity and dietary modification) are the first step in primary prevention (4). Regular moderate-intensity exercise helps lower blood pressure, blood lipids, and reduce the risk of heart attack and stroke. The “Chinese Guidelines for Hypertension Prevention and Treatment (2018 Revision)” recommends 120 min of moderate-intensity aerobic exercise per week (8).

In recent years, more studies have shown that appropriate aerobic exercise can effectively regulate the physiological and psychological aspects of patients with EH. Tai Chi, as an aerobic exercise, combines strength and flexibility with a balance of movement and stillness (9). The coordination of breathing, consciousness, and physical movements during Tai Chi enhances patient concentration, reduces sympathetic nervous tension, improves nervous system function, regulates blood pressure, and promotes sleep (10–12). Tai Chi, through its unique mechanisms of mind-quieting, body-relaxation, regulated breathing, and dantian cultivation, offers comprehensive benefits to cardiovascular health. It improves blood pressure and lipid levels through various pathways, including anti-oxidative stress (13), anti-inflammation (14), modulation of the autonomic nervous system (15), and enhancement of endothelial function (16). Existing research indicates that Tai Chi is more economical and safer compared to drug treatment, and it is the most effective method among all non-drug interventions (17, 18). Previous meta-analyses of Tai Chi on patients with essential hypertension generally focused only on its impact on blood pressure values (SBP, DBP), and there were few reports on blood lipids and body morphology (19, 20), different stages of hypertension, and intervention cycles. Dyslipidemia is another significant factor contributing to essential hypertension (EH) (21). The impact of different intervention cycles on the effect is huge.

To comprehensively evaluate the effects of Tai Chi on blood pressure and blood lipids in hypertensive patients, this study conducts a meta-analysis of RCTs involving Tai Chi interventions in hypertensive patients. This study will focus on examining the varying effects of Tai Chi interventions across different stages of hypertension, investigating the impacts of different intervention cycles on essential hypertension, then assessing its feasibility as a non-pharmacological treatment.

## 2 Methods

### 2.1 Protocol and registration

This systematic review was conducted according to the guidelines provided by the Preferred Reporting Items for Systematic Reviews and Meta-Analyses (PRISMA). The study protocol was registered with the International Prospective Register of Systematic Reviews (PROSPERO), under the registration number CRD42024538168.

### 2.2 Search strategy

We conducted comprehensive searches of three English-language databases (PubMed, Embase, Cochrane Library) and three Chinese-language databases (CNKI, Wan Fang, CBM) up to April 9, 2024. For PubMed, the search strategy included variations of “Tai Ji” such as “Tai Chi,” “taiji,” and “taijiquan,” combined with terms like “Hypertension,” “Blood Pressure,” or “High Blood Pressure.” The specific search strategy can be found in the Supplementary Materials.

### 2.3 Inclusion criteria

We employed the PICOS (Participants, Interventions, Comparisons, Outcomes, and Study design) framework to define our inclusion criteria (Table 1). Participants of all nationalities and genders were eligible, provided their average age was 40 years or above. The participants needed to meet the diagnostic criteria for hypertension (SBP ≥ 140 mm Hg and/or DBP ≥ 90 mm Hg) or prehypertension (SBP of 130–139 mm Hg and/or DBP of 80–89 mm Hg). The experimental group performed Tai Chi, either as a stand-alone intervention or combined with Medication treatment, such as oral nifedipine sustained-release tablets. The comparison group were those undergoing standard Western medical treatments, engaging in other forms of exercise, or served as controls. Additionally, studies had to be published with complete data.

**Table 1:** The inclusion criteria of PICOS.

PICOS components	Detail
Participants	40 ≤ Adults average age; hypertensive patient [The participants needed to meet the diagnostic criteria for hypertension (SBP ≥ 140 mm Hg and/or DBP ≥ 90 mm Hg) or prehypertension (SBP of 130 to 139 mm Hg and/or DBP of 80 to 89 mm Hg)].
Interventions	Taichi routine (common form)
Comparisons	Standardised western medical, sporting and non-sporting groups
Outcomes	Blood pressure (SBP, DBP); TC; TG; LDL; HDL; BMI; WC
Study designs	RCT

SBP, systolic blood pressure; DBP, diastolic blood pressure; TC, total cholesterol; TG, triglycerides; LDL, low-density lipoprotein; HDL, high-density lipoprotein; BMI, body mass index; WC, waist circumference; RCT, randomized controlled trial.

### 2.4 Exclusion criteria

The exclusion criteria were as follows: (1) lack of a control group; (2) duplicate studies, with preference given to the most comprehensive version; (3) non-original sources such as reviews or conference abstracts; (4) studies involving animal models; (5) participants with a history of myocardial infarction, chronic heart failure, arrhythmias, or other cardiovascular disorders; (6) experimental subgroups with fewer than 20 participants per group; (7) inadequate randomization methods; (8) studies classified as low quality.

### 2.5 Data extraction

The researchers systematically extracted data from all eligible publications. The extracted information included study authors, publication year, participant characteristics (health status, total sample size, mean age, and gender ratio), details of the intervention (frequency and duration), control measures, randomization and blinding methods, and reported outcomes. For studies with multiple observation points, only the final outcome data were considered.

### 2.6 Quality assessment

We assessed the methodological quality of the included studies using the Cochrane risk-of-bias tool (22). Two researchers independently evaluated each study, resolving any disagreements with a third reviewer. Seven domains were considered: random sequence generation (selection bias), allocation concealment (selection bias), blinding of participants and personnel (performance bias), blinding of outcome assessment (detection bias), incomplete outcome data (attrition bias), selective reporting (reporting bias), and other potential sources of bias (23). Studies were classified as low risk (five or more domains rated as low risk), moderate risk (three to four domains rated as low risk), or high risk (fewer than three domains rated as low risk) (24).

### 2.7 Statistical analysis

RevMan 5.3 was used to plot the quality assessment and the forest plots for all indicators, and Stata 14.0 was used for bias tests, sensitivity analyses, and funnel plots. The meta-analyses evaluated various participant parameters, including SBP, DBP, TC, TG, HDL, LDL, BMI, and WC. Mean effects and standard deviations (SD) were calculated using Stata 14.0, with 95% confidence intervals (CIs) were applied to all studies. Statistical significance was defined as *P* < 0.05. Effect sizes (ES) were used to evaluate the summary estimates. When there was no heterogeneity among the included studies (*P* > 0.05), a fixed-effects model was chosen for the Meta-analysis, and a random-effects model was applied when there was heterogeneity among the studies. Publication bias was assessed using Egger's test (25, 26), with significance set at *P* < 0.05.

## 3 Results

### 3.1 Study selection

**Figure 1:**
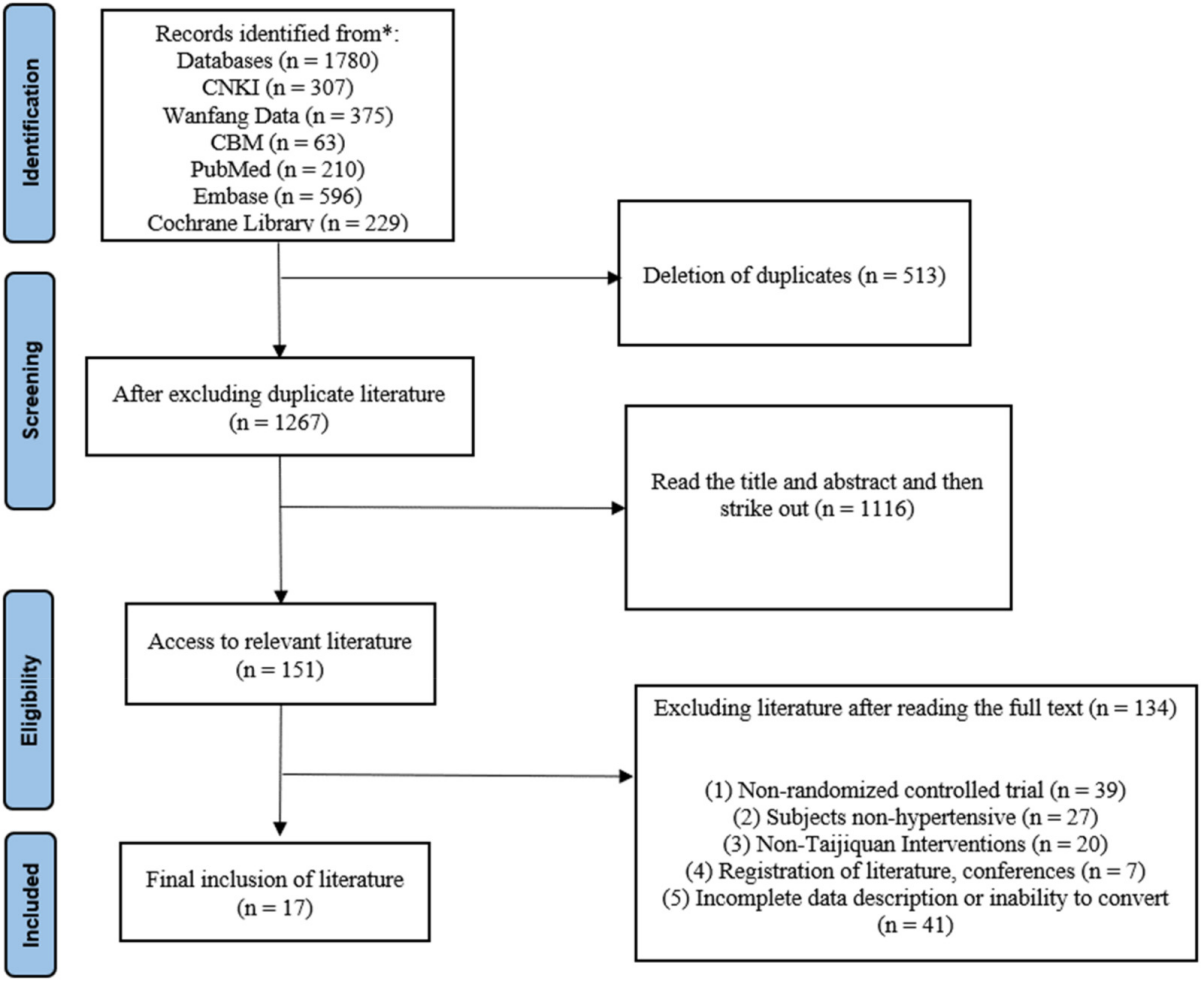
A PRISMA flow chart.

We searched Chinese and English databases and retrieved a total of 1,780 documents. First, NoteExpress literature management software was used to remove duplicates, literature reviews, meta-analyses, animal studies, and conference registrations. Titles, authors, publication years, and journals were then manually screened. Machine-based screening identified and excluded 513 duplicate papers. Further review of the remaining 1,267 abstracts led to the exclusion of studies for the following reasons: (1) not being randomized controlled trials, (2) participants not having hypertension, (3) interventions not involving Tai Chi, (4) being conference abstracts or registration papers, and (5) incomplete or unconvertible data. After a full-text review of 151 studies, 17 articles were deemed eligible for inclusion (Figure 1).

### 3.2 Characteristics of the included studies

Seventeen studies included Tai Chi as an independent intervention. Most studies were double-arm RCTs, although four studies used a three-arm RCT design. Of the double-arm trials, 11 compared the Tai Chi group to a blank control group, while the other two compared Tai Chi to alternative exercise programs, such as walking or aerobic exercise. Table 2 displays the detailed characteristics of the included studies. Among three-arm pilot studies, three included both blank control groups and groups engaging in other forms of exercise. Another study compared Tai Chi participants to a healthy population without involving additional exercise groups. In all the studies, the interventions spanned a minimum of 12 weeks, with session frequencies ranging from 1 to 10 times per week and session durations varying from 0.5 to 3 h. The most common intervention period was 12 weeks (observed in 12 studies), with a typical session frequency of 3–5 times per week.

**Table 2:** Characteristics of the included trials.

Trial	Sample size		Age		Intervention		Treatment		Outcomes
	Experimental group	Control group	Experimental group	Control group	Experimental group	Control group	Period (W)	Frequency	
Xiaobin Wang 2019 (27)	50(S) 20/30(M/W)	50(S) 22/28(M/W)	67.6 ± 4.5	67.4 ± 4.2	Twenty-four Patterns of Taijiquan	Routine treatment and care of hypertension	12	40–60 min, 3/w	①②③④⑤⑥
Lijuan Feng 2018 (28)	36(S) 19/17(M/W)	37(S) 14/23(M/W)	66.33 ± 4.74	67.51 ± 4.09	Taijiquan	1 h routine walk	12	1 h, 3/w	①②③④⑤⑥
Yongcai Zheng 2015 (29)	49(S) 20/29(M/W)	48(S) 19/29(M/W)	54.71 ± 5.43	55.77 ± 6.24	Twenty-four Patterns of Taijiquan	Nifedipine Sustained-release Tablets	12	40–60 min, 4–8/w	①②③④⑤⑥
Huijuan Xie 2014 (30)	25(S) 11/14(M/W)	25(S) 14/11(M/W)	60–70		Twenty-four Patterns of Taijiquan	General daily lifestyle interventions	12	1 h, 5/w	①②
Yami Cao 2024 (31)	40(S) 21/19(M/W)	40(S) 22/18(M/W)	66.98 ± 5.48	67.02 ± 5.46	Twenty-four Patterns of Taijiquan	Levoamlodipine Maleate Tablets	12	60 min, 1/d	①②
Shouxiao Ling 2018 (32)	98(S) 48/50(M/W)	100(S) 55/45(M/W)	52.35 ± 3.26	51.35 ± 4.21	Twenty-four Patterns of Taijiquan	General daily lifestyle interventions	12	45–60 min, 1–2/d	①②
Qiaoying Han 2010 (33)	30	28	62.12 ± 10.51		Twenty-four Patterns of Taijiquan	General daily lifestyle interventions	240	45–60 min, 1–2/d	①②
Xiaojun Wang 2011 (34)	30	30	50–70		Taijiquan Exercise Prescription	General daily lifestyle interventions	16	60 min, 5/w	①②
Zibo Shi 2017 (35)	30(S) 17/13(M/W)	30(S) 18/12(M/W)	43.26 ± 9.76	41.58 ± 9.12	Taijiquan	Low-salt, low-fat diet education	12	30 min, 4–5/w	①②
Jing Sun 2015 (11)	136(S) 19/117(M/W)	130(S) 29/101(M/W)	45–80		Taijiquan	Non-motor activity	48	Group activities 3 h/week and individual family exercises 2 h/week	①②③④⑤⑥⑦⑧
Xiao-Ling Shou 2019 (36)	98(S) 48/50(M/W)	100(S) 55/45(M/W)	52 ± 6.46(M), 51 ± 7.09(W)	52 ± 8.98(M) 51 ± 7.54(W)	Twenty-four Patterns of Taijiquan	General daily lifestyle interventions	12	40–90 min, 1–2/d	①②③④⑤⑥⑦
Aileen Wai Kiu Chan 2018 (37)	82(S) 32/50(M/W)	82(S) 38/44(M/W)	64.70 ± 7.59	65.13 ± 10.22	Twenty-four Patterns of Taijiquan	General daily lifestyle interventions	12	60 min, 2/w	①②③④⑤⑥⑦⑧
JEN-CHEN TSAI 2003 (10)	37(S) 19/18(M/W)	39(S) 19/20(M/W)	51.6 ± 16.3	50.5 ± 9.8	Taijiquan	General daily lifestyle interventions	12	50 min, 3/w	①②
Chunhua Ma 2018 (38)	79(S) 54/25(M/W)	79(S) 55/24(M/W)	70.24 ± 10.25	69.71 ± 10.84	Twenty-four Patterns of Taijiquan	Routine nursing of hypertension	24	60 min, 3–5/w	①②⑦⑧
Xinye Li 2024 (39)	173(S) 86/87(M/W)	169(S) 80/89(M/W)	48.4 ± 12.4	50.1 ± 11.4	Twenty-four Patterns of Taijiquan	Aerobic exercise	48	60 min, 4/w	①②③④⑤⑥⑦⑧
Zhi-Wei Yan 2022 (40)	49(S) 17/32(M/W)	47(S) 17/30(M/W)	59.31 ± 7.52	60.78 ± 7.21	Twenty-four Patterns of Taijiquan	General daily lifestyle interventions	12	60 min, 5/w	①②⑦
Bo Lin 2021 (41)	60(S) 36/24(M/W)	50(S) 29/21(M/W)	64.20 ± 4.04	63.80 ± 4.40	Twenty-four Patterns of Taijiquan	General daily lifestyle interventions	12	60 min, 3/w	①②

① SBP; ② DBP; ③ TC; ④ TG; ⑤ HDL; ⑥ LDL; ⑦ BMI; ⑧ (WC).

S, sum; M, male; W, female; W, week; d, day.

All 17 RCTS included were from China. With a total of 2,186 participants: 1,102 in the intervention group and 1,084 in the control group. Sample sizes ranged from 50 to 342 participants, with an average age exceeding 40 years. Two studies did not provide separate data for male and female participants; and all studies analyzed combined data for both sexes. None specifically investigated gender differences in the effects of Tai Chi. Two studies focused on working individuals with hypertension, and only one examined participants in a pre-hypertensive state.

Hypertension is primarily assessed through blood pressure measurements. All 17 articles included in this study evaluated blood pressure measurements (10, 11, 27–41). Dyslipidemia and hypertension are both risk factors for cardiovascular disease (42), and their co-occurrence may exacerbate arterial damage, with dyslipidemia influencing blood pressure. Seven studies specifically investigated lipid levels in hypertensive patients (11, 27–29, 36, 37, 39). BMI and WC are widely used to assess central obesity, a recognized risk factor for hypertension that can increase blood pressure through mechanisms such as insulin resistance and activation of the renin-angiotensin-aldosterone system (RAAS) (5). Six articles assessed participants' body composition and BMI levels (11, 36–40), while WC values were reported in four studies (11, 37–39).

### 3.3 Risk-of-bias assessment

**Figure 2:**
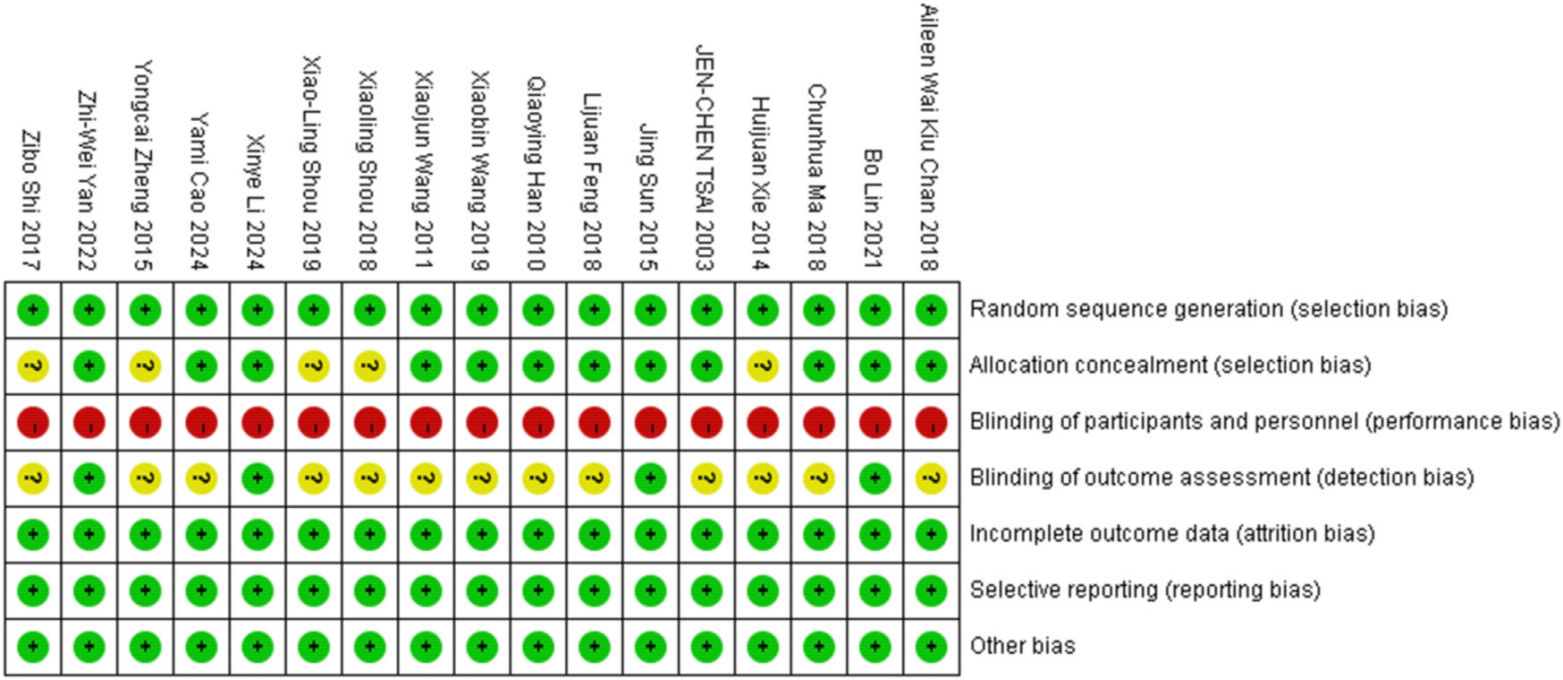
Biases of the included studies.

**Figure 3:**
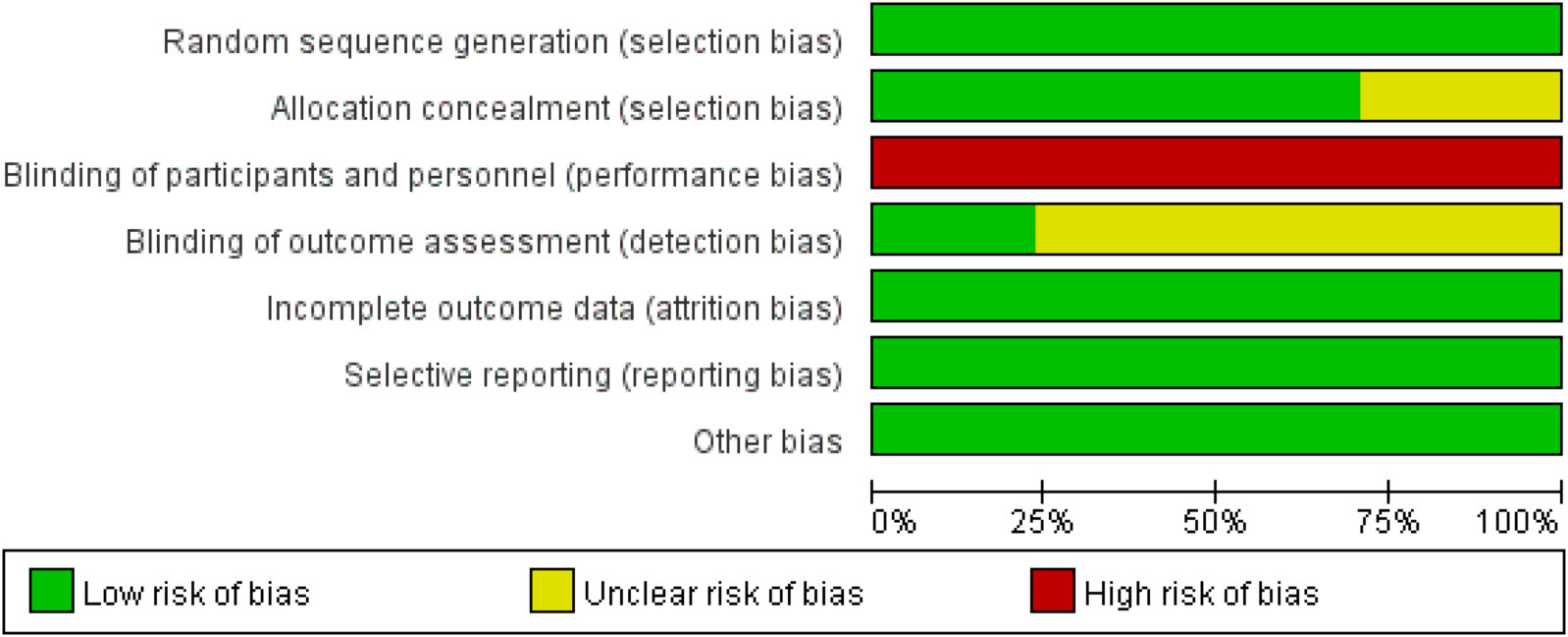
A summary of biases.

All 17 full papers were assessed for risk of bias using the Cochrane tool, with the quality evaluation results illustrated in Figures 2, 3. While all studies (100%) mentioned random allocation, only 13 (71%) studies provided details of specific random methods, such as the random number table method or computer-generated random numbers. The remaining four studies did not describe the allocation process. In Figure 3, five or more out of the seven dimensions indicate high quality, while fewer than five indicate medium to low quality. Twelve studies were classified as high quality. Five studies were rated as moderate quality due to unclear descriptions of allocation methods and participant blinding. Given the active participation required for Tai Chi, blinding of participants was not possible, leading to a high risk of performance bias in all studies. Sensitivity analysis and bias tests for all high-quality studies showed an Egger's test result of *P* = 0.441, which is greater than 0.05. This aligns with the overall findings, indicating the conclusions are reliable (details of the sensitivity analyses can be found in the Supplementary Material).

## 4 Effectiveness of Tai Chi

### 4.1 Tai Chi effect on SBP

**Figure 4:**
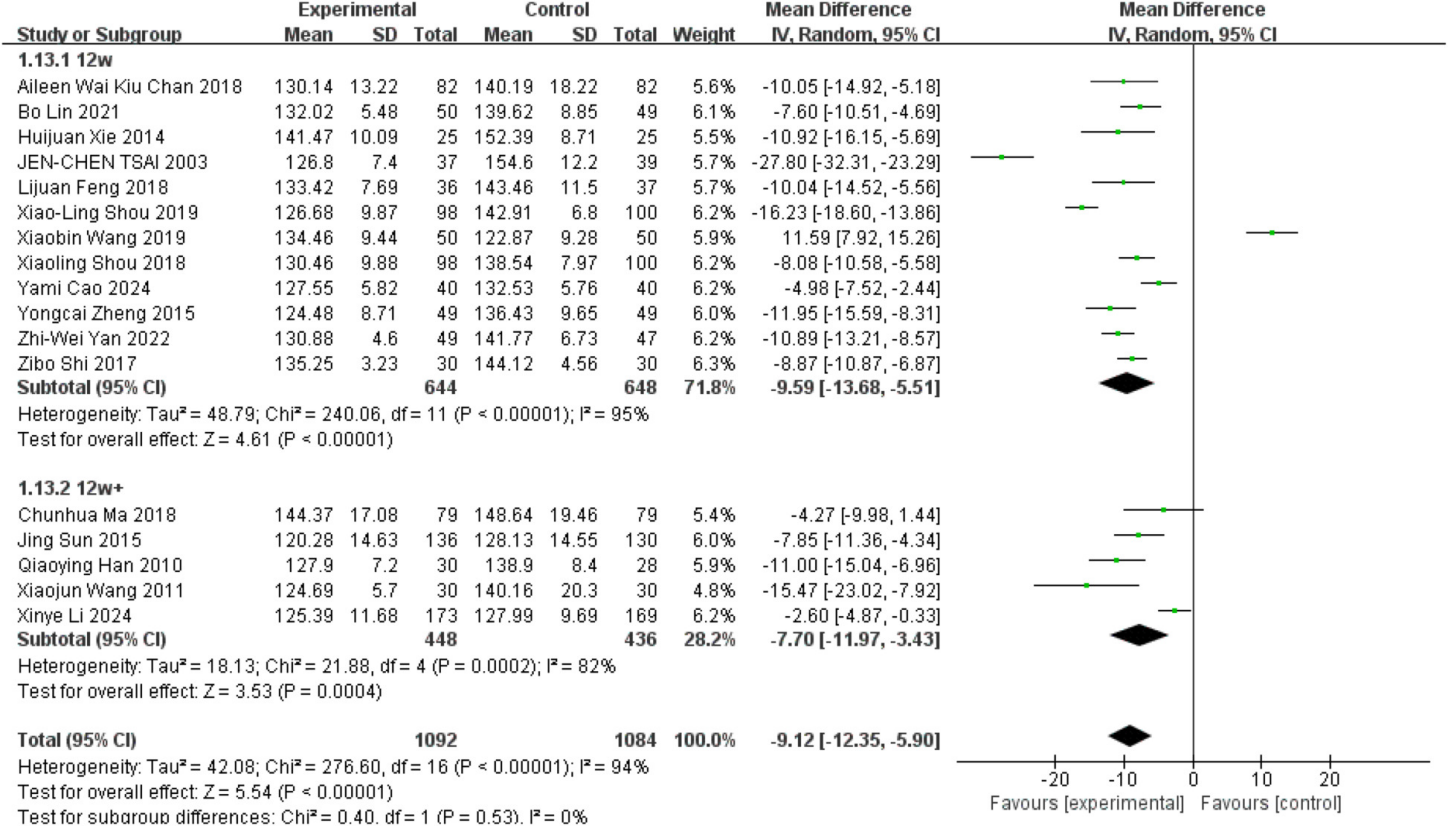
Effect of Tai Chi exercise on SBP levels in hypertensive patients (duration of intervention).

The meta-analysis focused on studies that used SBP as the primary outcome measure. Sixteen studies reported a significant decrease in SBP following the Tai Chi intervention (*p* < 0.05) (10, 11, 28–41), despite some studies showing contradictory results (27). Overall, the meta-analysis revealed that the decrease in SBP was notably higher in the Tai Chi group compared to control groups, although substantial heterogeneity was present (WMD = −9.12; 95% CI = −12.35 to −5.90; *Z* = 5.54, *P* = 0.000 < 0.05; *I*^2^ = 94%; Egger's test *P* = 0.685 > 0.05) (Figure 4). Full details of sensitivity analyses and bias assessments are provided in the Supplementary Appendix.

Tai Chi exercise is divided into long-term and short-term intervention cycles, defined as more than three months for long-term and less than three months for short-term interventions. To investigate potential differences in the effects of these intervention cycles on blood pressure in hypertensive patients, time was utilized as a subgroup variable. To further explore the effects of Tai Chi on SBP in hypertensive patients, studies were sub-grouped based on intervention duration: short-term (12 weeks) and long-term (more than 12 weeks). Twelve studies with a short-term intervention demonstrated a significant SBP reduction with Tai Chi (Figure 4, WMD = −9.59; 95% CI = −13.68 to −5.51; *Z* = 4.61, *P* = 0.00001 < 0.05; *I*^2^ = 95%; Egger's test *P* = 0.921 > 0.05) (10, 27–32, 35–37, 40, 41), Similarly, five studies with long-term intervention also showed a significant decrease in SBP (Figure 4, WMD = −7.70; 95% CI = −11.97 to −3.43; *Z* = 3.53, *P* = 0.0004 < 0.05; *I*^2^ = 82%; Egger's test, *P* = 0.153 > 0.05) (11, 33, 34, 38, 39).

**Figure 5:**
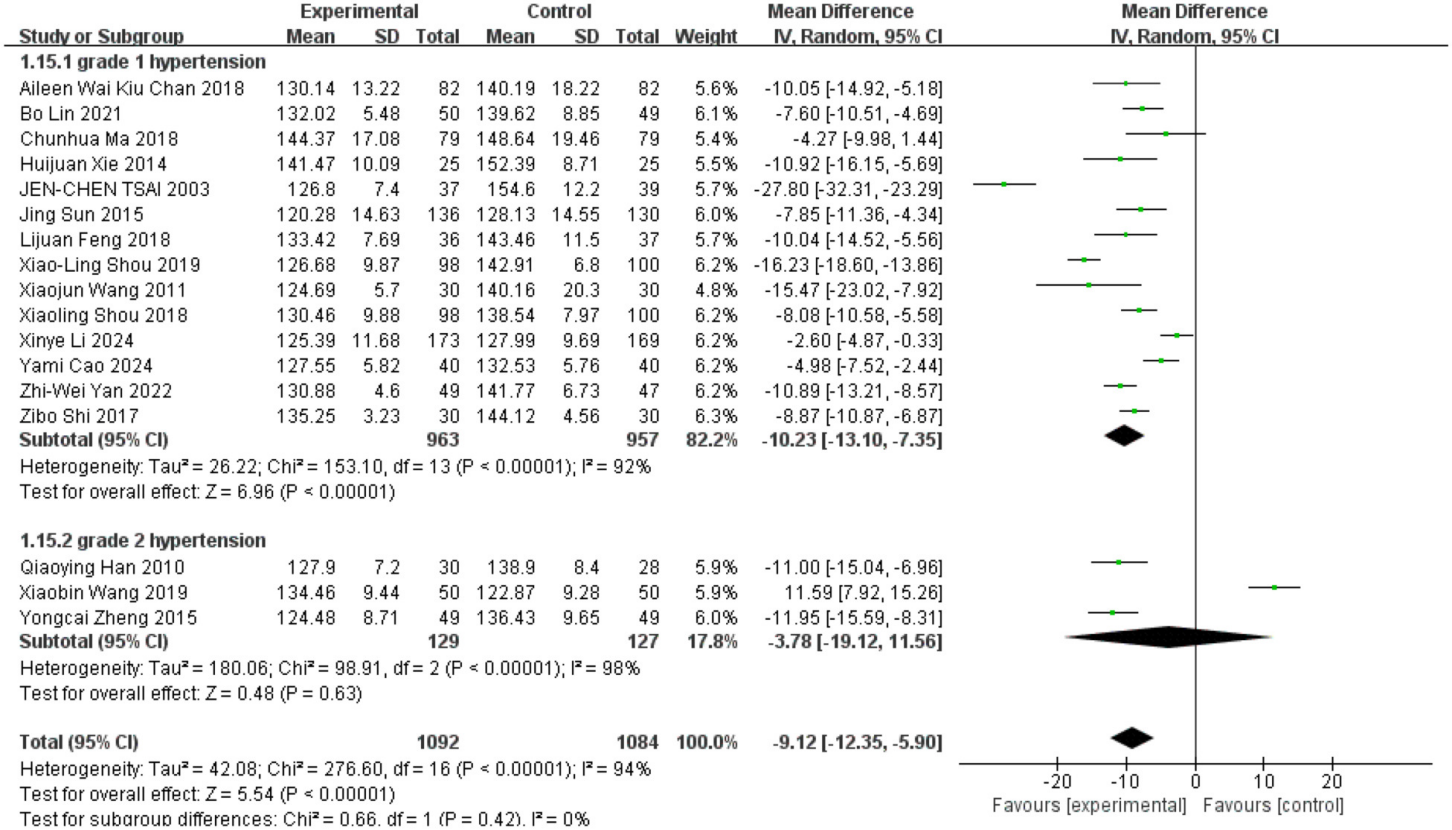
Effect of Tai Chi exercise on SBP levels in hypertensive patients (grade).

Hypertension is classified into three types: Grade I, Grade II, and Grade III. Grade I hypertension is defined as a systolic blood pressure (SBP) between 140 and 159 mmHg, or a diastolic blood pressure (DBP) between 90 and 99 mmHg. Grade II hypertension is defined as a SBP between 160 and 179 mmHg, or a DBP between 100 and 109 mmHg. Grade III hypertension is defined as a SBP of 180 mmHg or higher, or a DBP of 110 mmHg or higher (1). In studies examining the effects of Tai Chi on hypertension, these subtypes were used as grouping variables, classifying participants into Grade I and Grade II categories. Fourteen studies focused on Grade I hypertension showed that Tai Chi significantly reduced systolic blood pressure (SBP) (Figure 5, WMD = −10.23; 95% CI = −13.10 to −7.35; *Z* = 6.96, *P* = 0.00001 < 0.05; *I*^2^ = 92%; Egger's test *P* = 0.375 > 0.05) (10, 11, 28, 30–32, 34–41). Interestingly, three studies targeting Grade II hypertension indicated that while Tai Chi was able to reduce SBP, the difference was not statistically significant (Figure 5, WMD = −3.78; 95% CI = −19.12 to 11.56; *Z* = 0.48, *P* = 0.63 > 0.05; *I*^2^ = 98%; Egger's test *P* = 0.754 > 0.05) (27, 29, 33). There was no significant difference between the two groups. Through the one-by-one elimination method, it is found that Wang's (27) study in the Grade 2 hypertension group may be the source of its heterogeneity. After removing it, the analysis results of the Grade 2 hypertension group show that (WMD = −11.52; 95% CI = −14.23 to −8.82; *Z* = 8.35, *P* = 0.00001 < 0.05; *I*^2^ = 0%) (The figure is in the Supplementary Material).

### 4.2 Tai Chi effect on DBP

**Figure 6:**
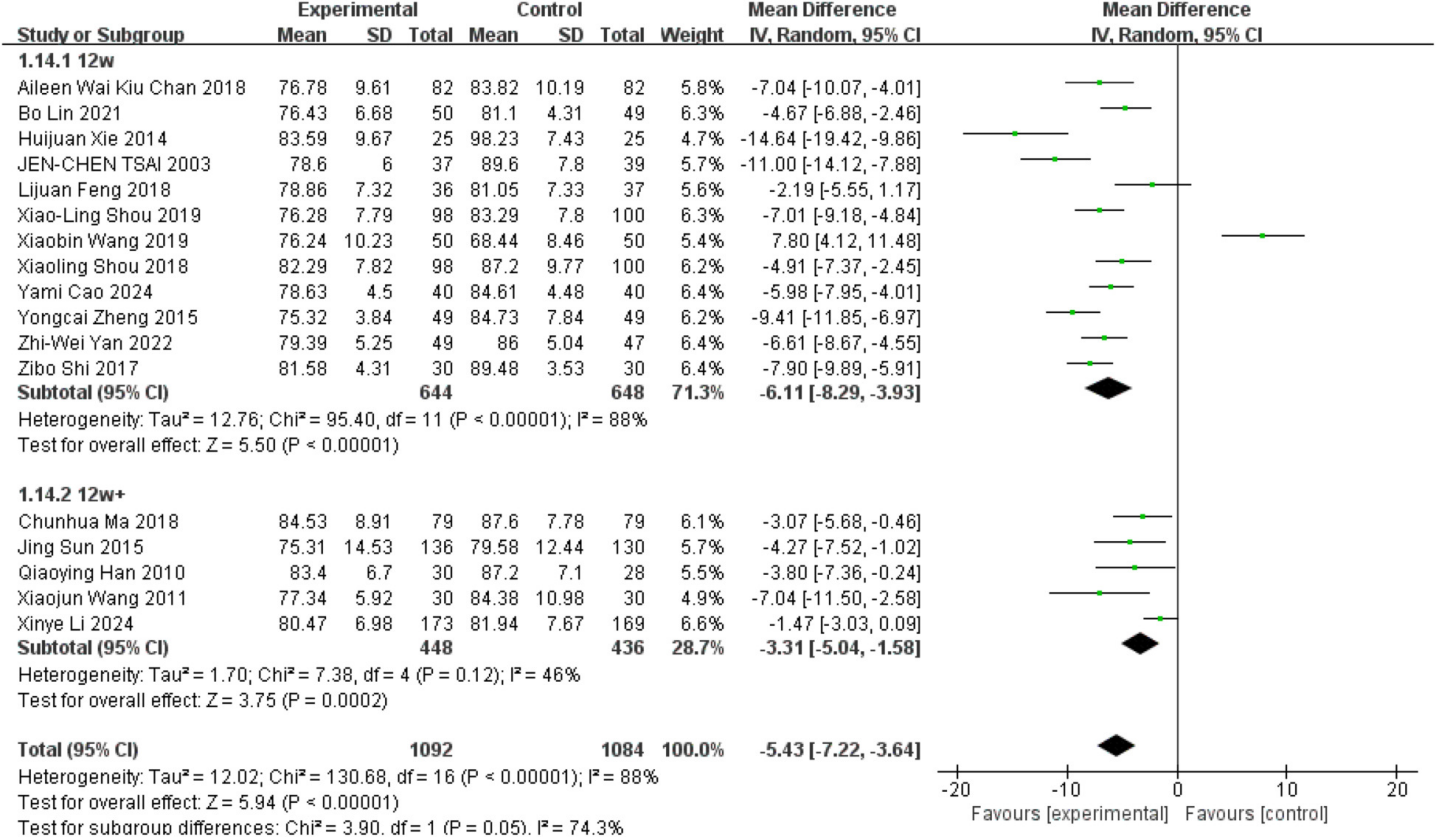
Effect of Tai Chi exercise on DBP levels in hypertensive patients (duration of intervention).

The meta-analysis included DBP values from all studies (10, 11, 27–41), with 16 studies reported a reduction in DBP following Tai Chi interventions lasting between 12 and 48 weeks (10, 11, 28–41), though some studies reported opposite effects (27). Overall, Tai Chi exercise significantly reduced participants' DBP levels compared to controls (Figure 6, WMD = −5.43; 95% CI = −7.22 to −3.64; *Z* = 5.94, *P* = 0.000 < 0.05; *I*^2^ = 88%; Egger's test *P* = 0.67 > 0.05).

Subgroup analyses revealed a highly significant reduction in DBP among participants engaged in Tai Chi for 12 weeks compared to controls (Figure 6, WMD = −6.11; 95% CI = −8.29 to −3.93; *Z* = 5.50, *P* = 0.00001 < 0.05; *I*^2^ = 88%; Egger's test, *P* = 0.689 > 0.05). Additionally, Tai Chi participants demonstrated significantly lower DBP levels compared to controls over intervention durations exceeding 12 weeks (Figure 6, WMD = −3.31; 95% CI = −5.04 to −1.58; *Z* = 3.75, *P* = 0.0002 < 0.05; *I*^2^ = 46%; Egger's test, *P* = 0.005).

**Figure 7:**
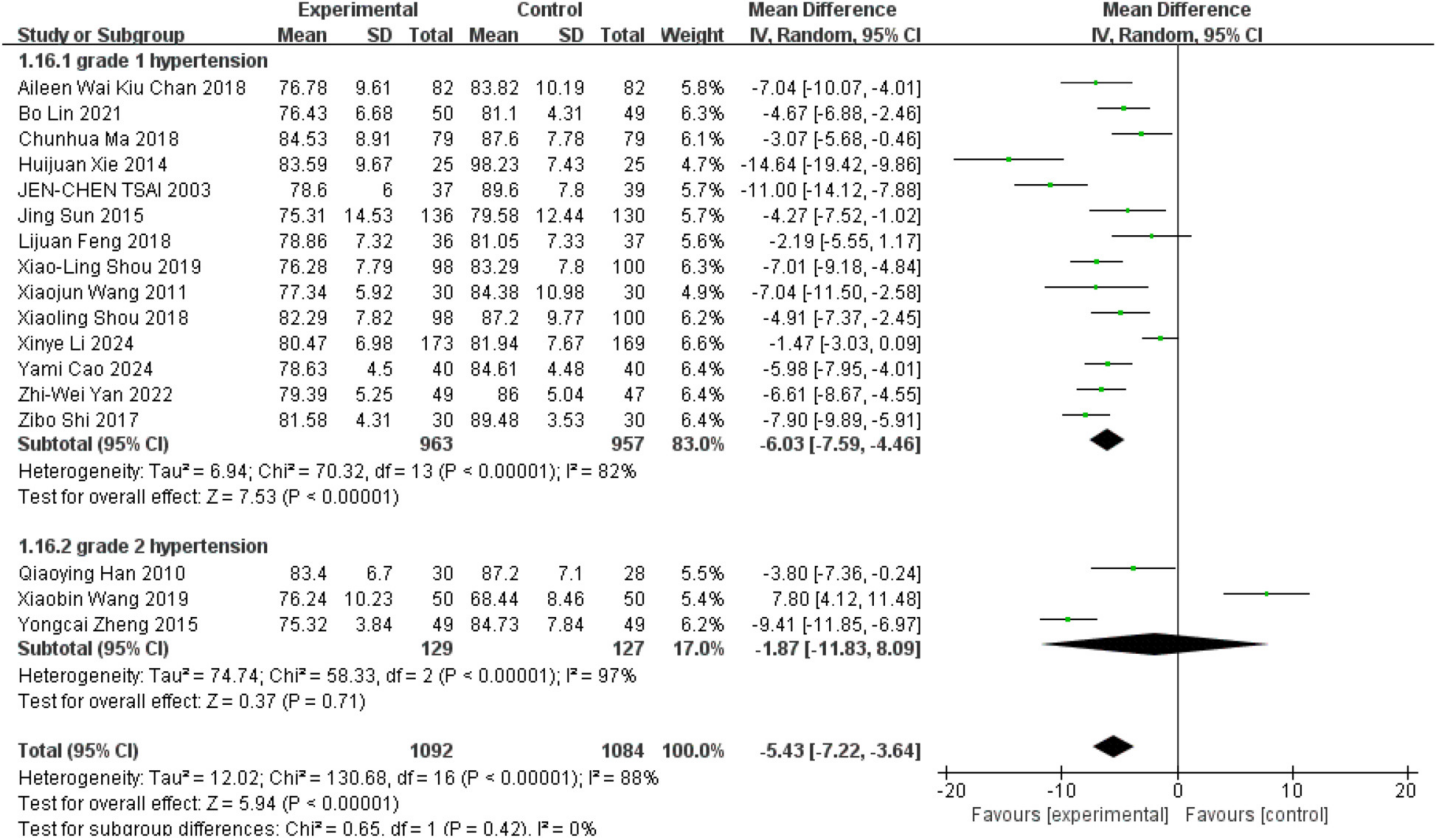
Effect of Tai Chi exercise on DBP levels in hypertensive patients (grade).

In studies examining the effects of Tai Chi on hypertension, these subtypes were used as grouping variables, classifying participants into Grade I and Grade II categories. Fourteen studies focused on Grade I hypertension showed that Tai Chi significantly reduced diastolic blood pressure (DBP) (Figure 7, WMD = −6.03; 95% CI = −7.59 to −4.46; *Z* = 7.53, *P* = 0.00001 < 0.05; *I*^2^ = 82%; Egger's test *P* = 0.092 > 0.05) (10, 11, 28, 30–32, 34–41). Similarly, three studies of class II hypertension showed that although Tai Chi was able to reduce diastolic blood pressure, the difference was not statistically significant (Figure 7, WMD = −1.87; 95% CI = −11.83 to 8.09; *Z* = 0.37, *p* = 0.71 > 0.05; *I*^2^ = 97%; Egger's test *p* = 0.35 > 0.05) (27, 29, 33).

### 4.3 Tai Chi effects on TC

**Figure 8:**
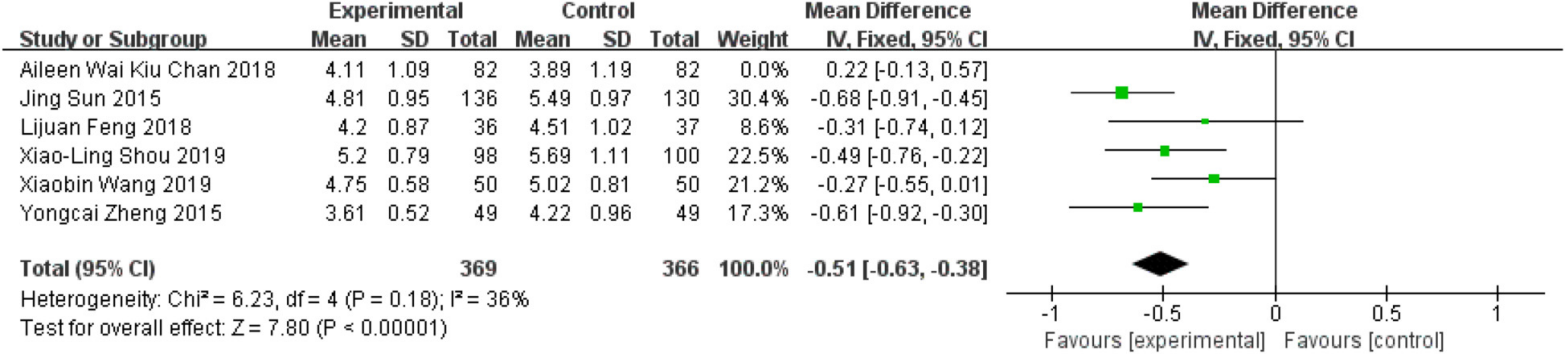
Forest plot comparing TC in Tai Chi and all controls.

Six studies evaluated the effect of Tai Chi on TC levels (11, 27–29, 36, 37). The meta-analysis demonstrated that Tai Chi exercise significantly reduced TC levels compared to the control groups (WMD = −0.37; 95% CI = −0.62 to −0.12; *Z* = 2.91, *P* = 0.004 < 0.05; *I*^2^ = 76%; Egger's test, *P* = 0.213 > 0.05) (The chart is attached to the Supplementary Material). Using one-by-one elimination method, it is found that the source of heterogeneity is Aileen's research (37) (Figure 8, WMD = −0.51; 95% CI = −0.63 to −0.38; *Z* = 7.8, *P* = 0.00001 < 0.05; *I*^2^ = 36%; Egger's test, *P* = 0.377 > 0.05).

### 4.4 Tai Chi effects on TG

**Figure 9:**
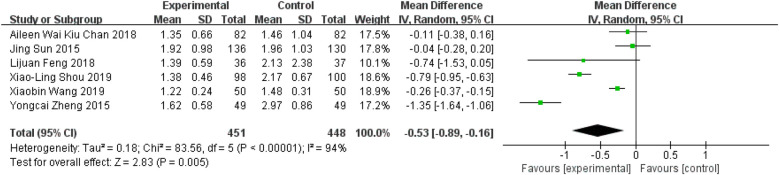
Forest plot comparing TG in TC and all controls.

These six studies reported participants' TG levels (11, 27–29, 36, 37). The meta-analysis revealed a notable decrease in TG levels among Tai Chi participants in comparison to the control group (Figure 9, WMD = −0.53; 95% CI = −0.89 to −0.16; *Z* = 2.83, *P* = 0.005 < 0.05; *I*^2^ = 94%; Egger's test, *P* = 0.606 > 0.05).

### 4.5 Tai Chi effects on LDL

**Figure 10:**
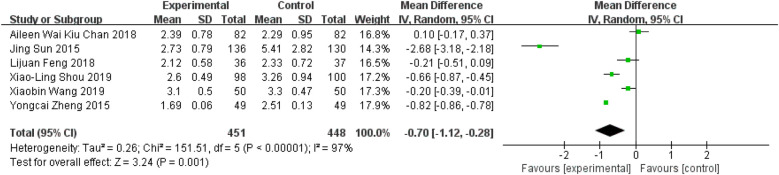
Forest plot comparing LDL in TC and all controls.

Six studies reported data on participants' LDL levels (11, 27–29, 36, 37). The meta-analysis showed that Tai Chi exercise had a highly significant effect on reducing LDL levels compared to the control group (Figure 10, WMD = −0.70; 95% CI = −1.12 to −0.28; *Z* = 3.24, *P* = 0.001 < 0.05; *I*^2^ = 97%; Egger's test, *P* = 0.522 > 0.05).

### 4.6 Tai Chi effects on HDL

**Figure 11:**
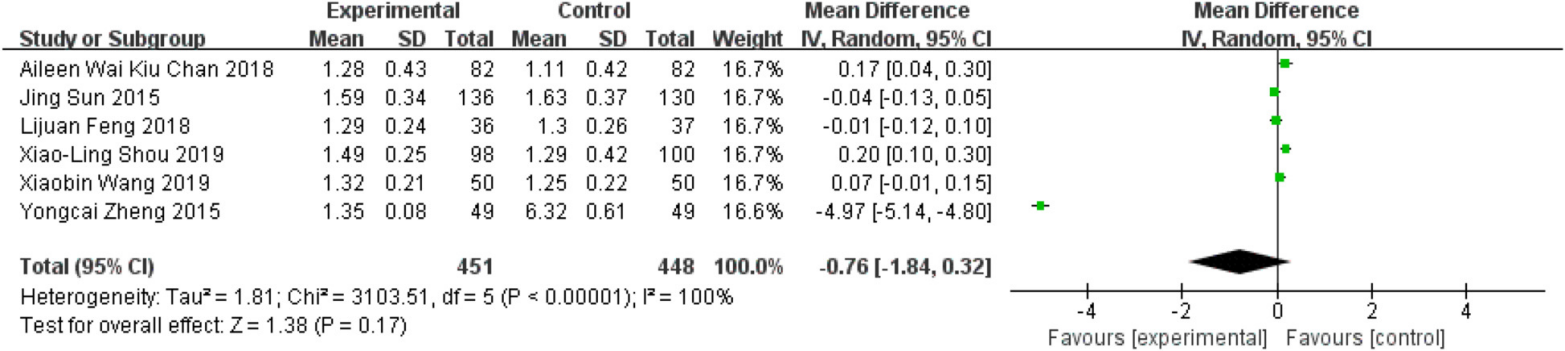
Forest plot comparing HDL in TC and all controls.

Six studies reported data on participants' HDL levels (11, 27–29, 36, 37). The meta-analysis showed that participants in the Tai Chi exercise group exhibited non-significant improvements in HDL levels compared to the control group (Figure 11, WMD = −0.76; 95% CI = −1.84 to 0.32; *Z* = 1.38, *P* = 0.17 > 0.05; *I*^2^ = 100%; Egger's test, *P* = 0.089 > 0.05).

### 4.7 Tai Chi effects on BMI

**Figure 12:**
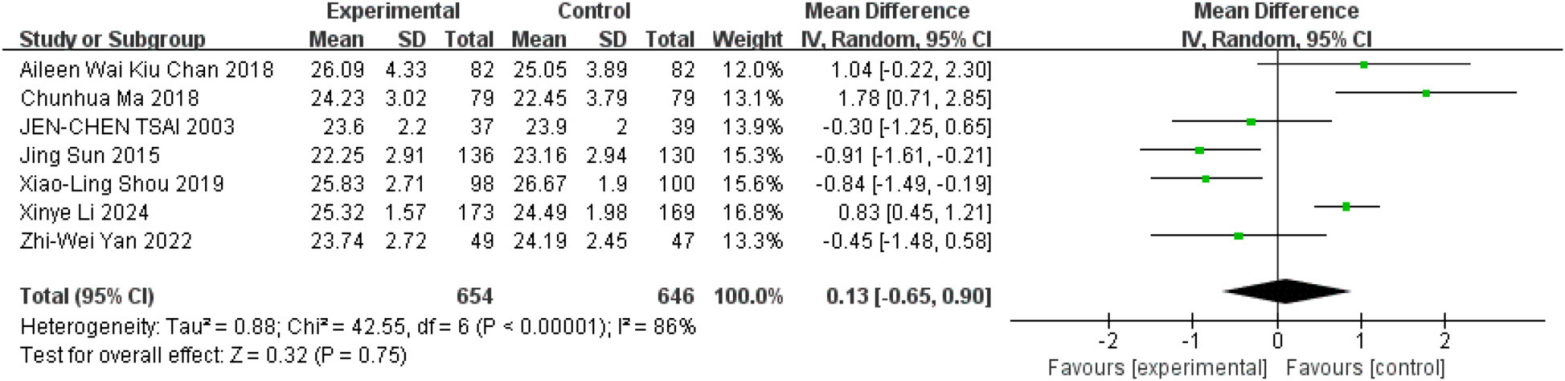
Forest plot comparing BMI in TC and all controls.

A total of seven studies reported data on participants' BMI (10, 11, 36–40). The meta-analysis revealed that Tai Chi exercise did not have a statistically significant effect on BMI levels compared to the control group (Figure 12, WMD = 0.13; 95% CI = −0.65 to 0.90; *Z* = 0.32, *P* = 0.75 > 0.05; *I*^2^ = 86%; Egger's test, *P* = 0.626 > 0.05).

### 4.8 Tai Chi effects on WC

**Figure 13:**
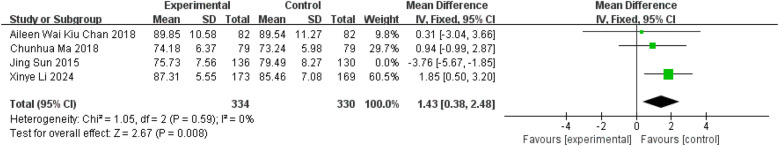
Forest plot comparing WC in TC and all controls.

Four studies reported data on participants' WC levels (11, 37–39). The meta-analysis revealed that Tai Chi exercise did not significantly improve WC levels compared to the control group (WMD = −0.16; 95% CI = −2.88 to 2.57; *Z* = 0.11, *P* = 0.91 > 0.05; *I*^2^ = 87%; Egger's test, *P* = 0.656 > 0.05) (The chart is attached to the Supplementary Material). By means of one-by-one elimination method, it is found that the source of heterogeneity is Sun's research (11) (Figure 13, WMD = 1.43; 95% CI = 0.38 to 2.48; *Z* = 2.67, *P* = 0.008 < 0.05; *I*^2^ = 0%; Egger's test, *P* = 0.246 > 0.05).

## 5 Discussion

This systematic review examined randomized controlled trials (RCTs) investigating the effects of Tai Chi exercise on patients with essential hypertension, with separate subgroup analyses based on the duration of the intervention and the type of hypertension. The results indicated that practicing Tai Chi significantly reduced participants' blood pressure (SBP, DBP), and blood lipids (TC, TG, LDL). However, high density lipoprotein (HDL) and body mass index (BMI) did not change significantly, and waist circumference (WC) showed an increasing trend.

Hypertension is classified into essential and secondary types. Secondary hypertension arises due to specific underlying conditions and accounts for only a small percentage of hypertensive patients, while the majority of individuals with hypertension have primary hypertension (1). The pathogenesis of essential hypertension involves a complex interaction of genetic, environmental, and aging factors (43–45), which contribute to the development of the condition by disrupting the cardiovascular regulatory system. This disruption leads to increased vascular resistance (46), often accompanied by alterations in the renin-angiotensin-aldosterone system (RAAS) and the endothelin system (46–48). It is currently believed that nitric oxide (NO), synthesized and released by vascular endothelial cells, plays an important protective role in the cardiovascular system. NO helps maintain blood pressure, prevents endothelial damage, reduces blood viscosity, and decreases the formation of pathological blood clots and atherosclerotic plaques (49). The imbalance between NO and endothelin-1 (ET-1), along with the dysfunction of endothelium-dependent vasodilation caused by endothelial dysfunction, is a key mechanism in the development of hypertension (50, 51). Beyond these direct contributors, hypertension is also associated with other risk factors such as dyslipidemia, impaired glucose tolerance, and type 2 diabetes mellitus, which further elevate cardiovascular risk (52). The research reported a decrease in blood pressure values after Tai Chi intervention, which is consistent with the results of previous studies (53). Moreover, Subgroup analysis of different control measures (other exercise and medication) revealed that Tai Chi resulted in an average reduction of 4.51 mmHg in SBP compared to other exercises and 9.46 mmHg compared to medications (Supplementary Figure S16). Regarding DBP, Tai Chi led to an average reduction of 5.05 mmHg compared to other exercises and 5.61 mmHg compared to medications (Supplementary Figure S17). When patients with essential hypertension (EH) practice Tai Chi, its mechanisms of mind-quieting and body-relaxing, breathing and exhaling, and dantian drumming help one enter an alpha-wave state, achieve relaxation of the whole body and brain, reduce the patient's state of stress (54), regulate the autonomic nervous system, especially promote the dilatation of small arteries throughout the body, alleviate the sclerosis of the arterial walls by enhancing the activity of the parasympathetic nerves and decreasing sympathetic nervous tension, and increasing the release of NO, thereby reducing blood pressure (10, 54, 55). In addition, Tai Chi, as a group exercise, reduces stress and enhances emotional well-being through social support, which further contributes to the reduction of blood pressure (56).

Dyslipidemia are independent risk factors for hypertension, with studies showing that increased levels of TC, low-density lipoprotein cholesterol (LDL-C), and TG significantly increase the risk of hypertension (57, 58). Elevated levels of TC and TG contribute to atherosclerosis, a condition that leads to the hardening and thickening of the vessel walls, narrowing the lumen, and increasing peripheral resistance, ultimately resulting in elevated blood pressure (58). This study reports the decline of partial blood lipids function (TC, TG, LDL) after Tai Chi intervention. However, in contrast to previous studies (59), the impact of Tai Chi on HDL levels was not statistically significant. There was considerable variation in HDL outcomes across studies, with Zheng et al. reporting a decrease of 4.97 mmol/L in HDL levels (29). After excluding this outlier, the remaining studies showed an average increase of 0.235 mmol/L in HDL levels, though the change was not statistically significant. Overweight or obesity is directly linked to hypertension (60). Individuals with abdominal obesity are at a heightened risk of developing hypertension (61). BMI and WC are correlated with the prevalence of hypertension and can effectively predict its occurrence (62). The results of this study revealed that the effects of Tai Chi intervention on BMI and WC were contrary to those of previous studies (63, 64). Initially, the results of this study showed a slight decrease in WC levels, but the heterogeneity was relatively high. There may be considerable heterogeneity in the study by Sun et al. (11), which demonstrated a greater effect of Tai Chi exercise on WC compared to the other studies included in the analysis. After excluding this study, there was a general trend of elevated WC levels in hypertensive patients following Tai Chi intervention. Our findings show that Tai Chi practice has little effect on this, which could be due to not having good control of the diet (65), individual differences (66), or measurement bias (67).

Notably, the effect of Tai Chi practice was more pronounced at 12 weeks compared to long-term practice, though long-term practice still showed significant benefits when compared to the control group. This finding aligns with the minimum intervention duration (12 weeks) suggested by the 8th Joint National Committee study (68), and, in conjunction with our findings, underscores the importance of 12 weeks as a benchmark for the duration of effective Tai Chi intervention in patients with essential hypertension. It might because the 12-week intervention cycle brought the practice effect of Tai Chi to its peak. However, the effect of cycles over 12 weeks was still more effective than blank control group, but it was lower than the peak. This provides a basis for the fact that Tai Chi intervention for essential hypertension can take effect after 12 weeks. In addition, Tai Chi intervention was found to be more effective in patients with Grade 1 hypertension (*P* < 0.05) than in those with Grade 2 hypertension (*P* > 0.05), although the difference between the two groups was not statistically significant. The underlying mechanism may be related to the fact that Grade 1 hypertension can typically be managed through lifestyle changes, whereas Grade 2 hypertension often requires a combination of medications to effectively control blood pressure. In this study, participants continued their usual medication regimen, and the effect of Tai Chi on Grade 1 essential hypertension was more pronounced. However, while the intervention appeared to have a slight downward effect on Grade 2 hypertension, the result was not statistically significant and warrants further investigation in future studies. Wang's research showed opposite results to the other two studies. When Wang's study was excluded (27), Tai Chi showed similar effectiveness in both groups, with no significant difference between them. This may be due to the limited number of studies in the Grade 2 hypertension subgroup and the small sample size. This study demonstrated strong heterogeneity in several indicators such as SBP and DBP. After analyzing the original literature, it was found that this might be due to the duration, frequency, cycle of the intervention and the age of participants. The longest intervention period was as long as 5 years, and the shortest was 12 weeks. Each intervention lasted 30–90 min. The frequency was as high as 1–2 times a day and at least 3 times a week. The age range varied from 40 to 80 years old. Although these intervention level classifications have enriched our results to some extent, they inevitably have a certain impact on the reliability of the results. We have tried our best to eliminate this impact by using random effects models, sensitivity analysis and other methods. Therefore, it is suggested that a unified and standardized Tai Chi intervention plan should be established in future research. To enhance the validity and applicability of science.

Tai Chi may serve as both a physical and mental exercise. Through its unique mechanism of calming the mind and relaxing the body, it can stimulate the functions of internal organs, regulate qi, blood, and bodily fluids, and help the body achieve a state of relaxation and calmness. Additionally, it has been shown to reduce markers of oxidative stress (69), improve psychological well-being (70), lower blood lipid levels (71), and promote lipid metabolism (72), thereby stabilising blood pressure (73). However, the specific underlying mechanisms require further investigation.

## 6 Limitation

There exist several limitations in our investigation. Firstly, our meta-analysis revealed substantial heterogeneity across studies for several outcomes (*I*^2^ > 80%). We used the stepwise exclusion method and found that the heterogeneity was not caused by any single study. Furthermore, the randomisation procedures used in some of the studies were not publicly available, which may have led to selection bias. Third, because of the unique format of the Tai Chi exercise intervention, it was not possible to blind the assessors, which may have led to the presence of performance bias. Forth, the level of quality of the five studies included in the analysis could be considered “acceptable”, which may have influenced the results to some extent (Supplementary Figures S14, S15). Finally, the studies included were limited to English and Chinese literature, potentially introducing bias by excluding other languages. There were variations in the form, style, frequency, and duration of Tai Chi across the studies. It is suggested that a unified and standardized Tai Chi intervention plan should be established in future research. To enhance the validity and applicability of science.

## 7 Conclusion

This study systematically evaluated the effects of Tai Chi on blood pressure, blood lipids, and body composition in patients with essential hypertension, exploring its feasibility as a non-pharmacological treatment. The findings revealed that Tai Chi effectively lowers levels of SBP, DBP, TC, TG, and LDL. Its efficacy surpasses that of other exercise modalities, with the most pronounced improvements observed at the 12-week mark. In addition, it was observed that Tai Chi has a better blood pressure-lowering effect on grade 1 hypertension than on grade 2 hypertension. These findings support the use of Tai Chi as a scientifically validated exercise rehabilitation tool in clinical settings and suggest avenues for further research. Future research should address several priorities. Firstly, it is important to explore why three-month interventions are the most effective. Additionally, further research is needed to explore the effects of Tai Chi intervention on different stages of hypertension. This will help validate the findings and address potential bias in the results due to the limited literature on type 2 hypertension in this study. Secondly, large-scale randomized controlled trials should be conducted to develop standardized protocols, eliminating potential bias from variations in Tai Chi forms, styles, frequency, and duration. Thirdly, research should evaluate Tai Chi 's effectiveness across different populations and health conditions, with particular attention to cultural adaptation and acceptance for global implementation. Finally, more detailed studies are needed to explore whether the benefits of Tai Chi vary by gender and age group.

## Data Availability

The datasets presented in this study can be found in online repositories. The names of the repository/repositories and accession number(s) can be found in the article/Supplementary Material.
