# Supplementary material for: An RCT META analysis based on the efficacy of Tai Chi exercise therapy on blood pressure and blood lipids in patients with essential hypertension
Source: Front Cardiovasc Med. 2025 Aug 12;12:1506912. doi: 10.3389/fcvm.2025.1506912 (PMC12378830; doi:10.3389/fcvm.2025.1506912)
Supplement: Supplementary file 1 [file Datasheet1.pdf]

Catalogues

attachment (email) ... 2
SBP ... 2
    All - Sensitivity Tests ... 2
    All - bias testing ... 3
    12W - Sensitivity Tests ... 3
    12W - bias testing ... 4
    12W or more - Sensitivity Tests ... 4
    12W or more - bias testing ... 5
    Grade I hypertension - Sensitivity Tests ... 5
    Grade I hypertension - bias testing ... 6
    Grade II hypertension - Sensitivity Tests ... 6
    Grade II hypertension - bias testing ... 7
    Grade II hypertension -Sensitivity analysis after elimination ... 7
    Grade II hypertension - Culled funnel diagram ... 8
DBP ... 8
    All - Sensitivity Tests ... 8
    All - bias testing ... 9
    12W - Sensitivity Tests ... 9
    12W - bias testing ... 10
    12W or more - Sensitivity Tests ... 10
    12W or more - bias testing ... 11
    Grade I hypertension - Sensitivity Tests ... 11
    Grade I hypertension - bias testing ... 12
    Grade II hypertension - Sensitivity Tests ... 12
    Grade II hypertension - bias testing ... 13
TC ... 13
    All - Sensitivity Tests ... 13
    Sensitivity analysis after elimination ... 14
    All - bias testing ... 14
    Culled funnel diagram ... 15
TG ... 15
    All - Sensitivity Tests ... 15
    All - bias testing ... 16
HDL ... 16
    All - Sensitivity Tests ... 16
    All - bias testing ... 17
LDL ... 17
    All - Sensitivity Tests ... 17
    All - bias testing ... 18
BMI ... 18
    All - Sensitivity Tests ... 18
    All - bias testing ... 19

WC 19
All - Sensitivity Tests 19
Sensitivity analysis after elimination 20
All - bias testing 20
Culled funnel diagram 21
High-quality research sensitivity analyses 21
Search strategy 22
Meta-analysis results graph 23

attachment (email)

SBP

All - Sensitivity Tests

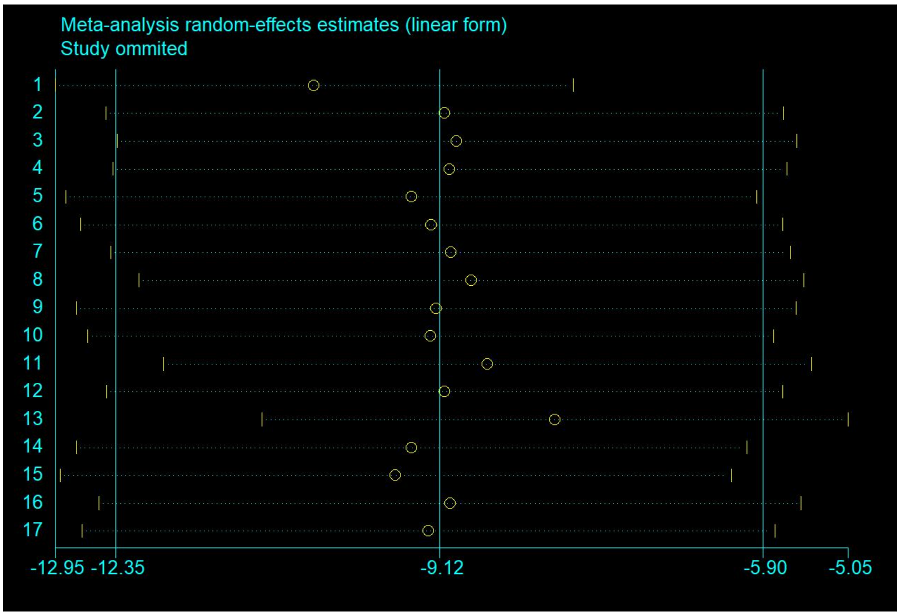

All - bias testing

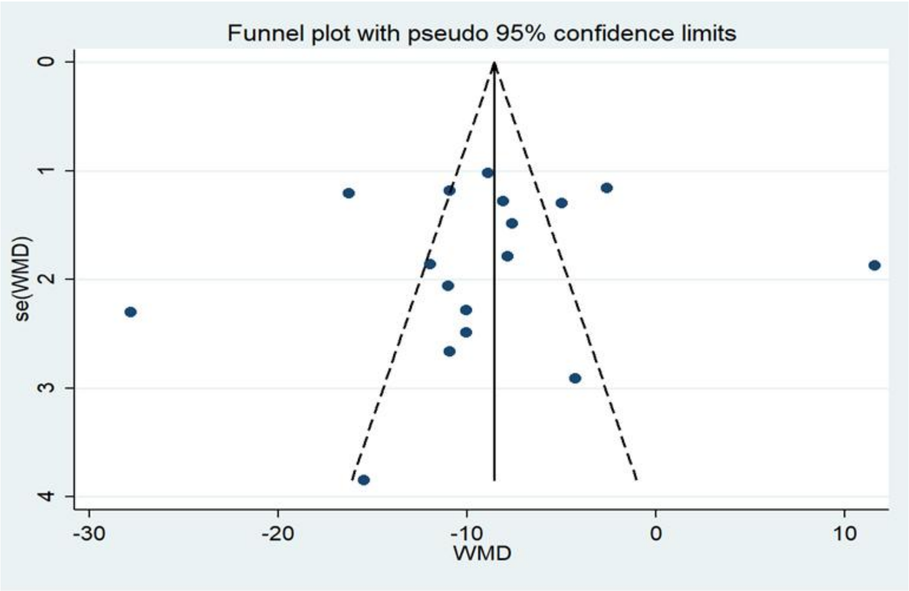

12W - Sensitivity Tests

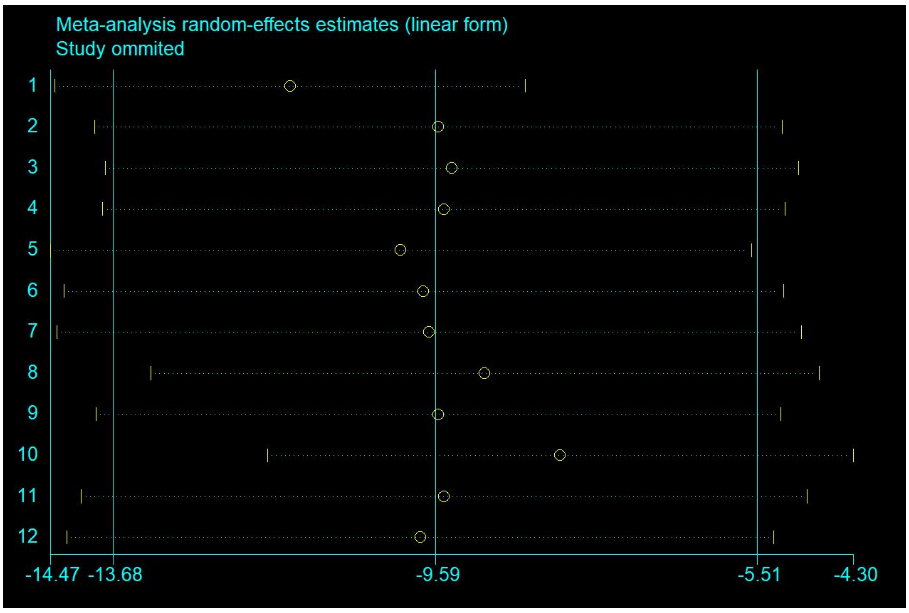

12W - bias testing

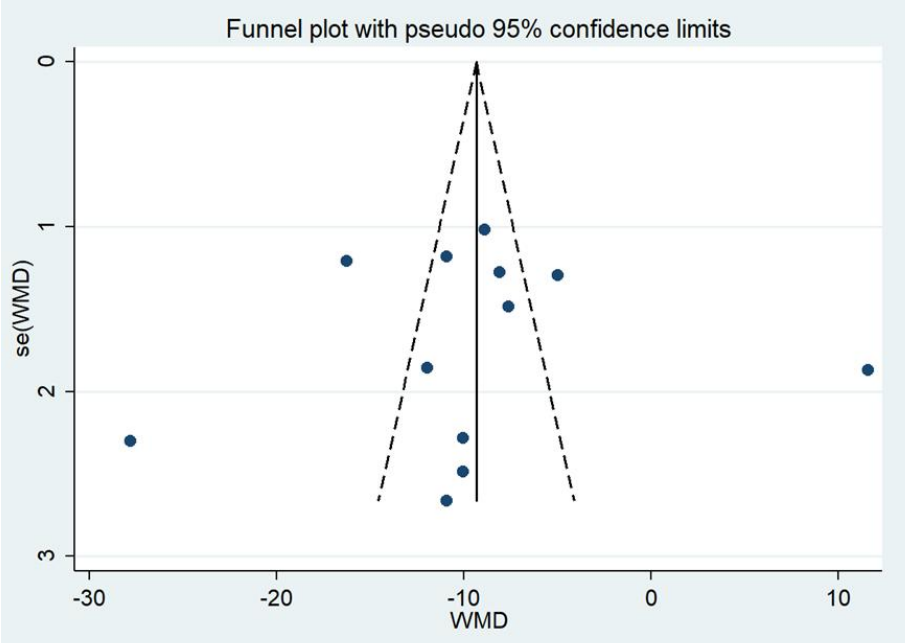

12W or more - Sensitivity Tests

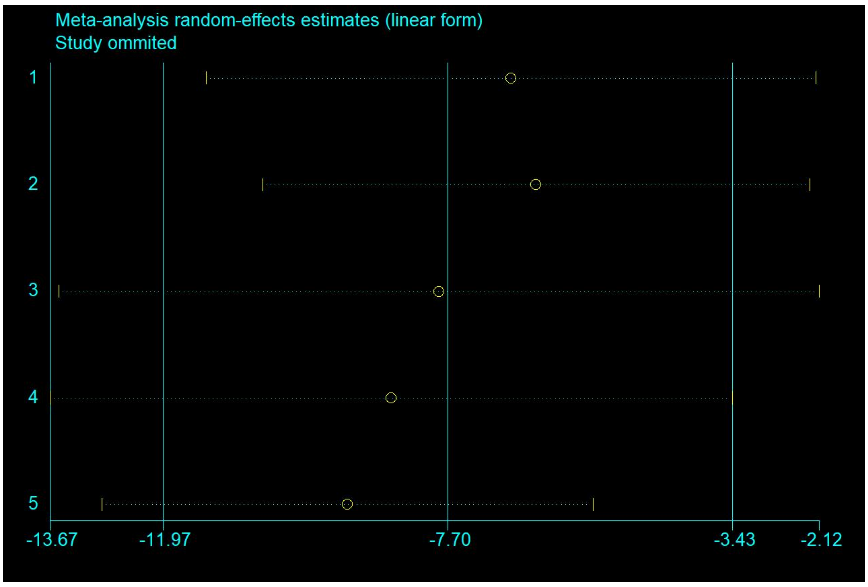

12W or more - bias testing

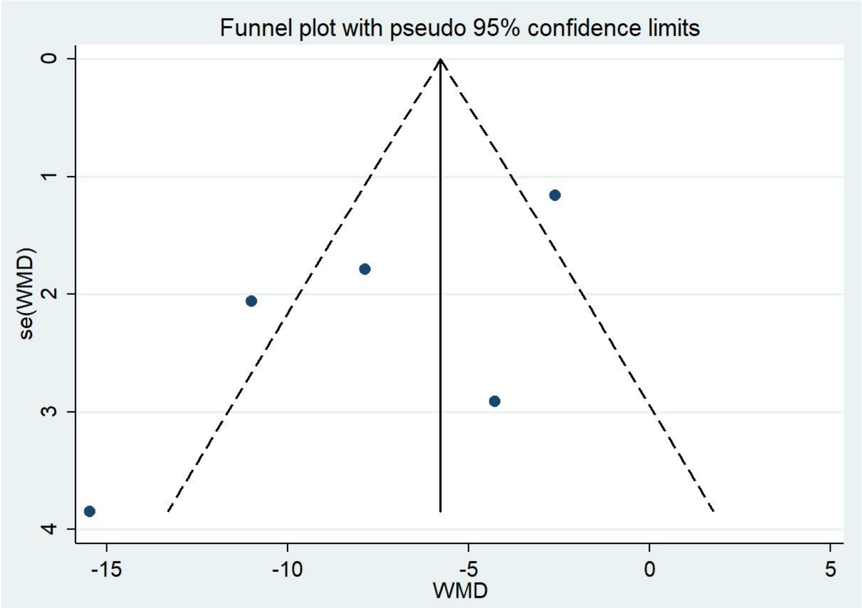

Grade I hypertension - Sensitivity Tests

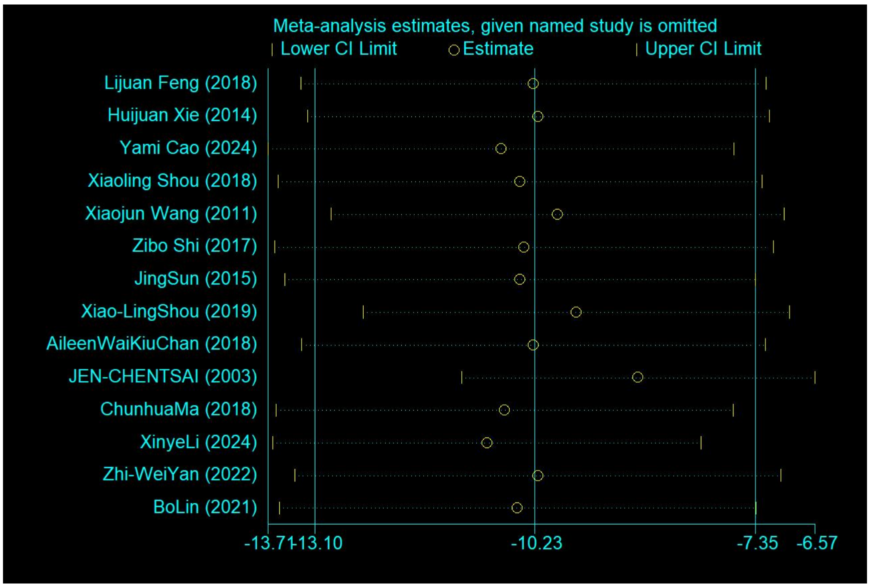

Grade I hypertension - bias testing

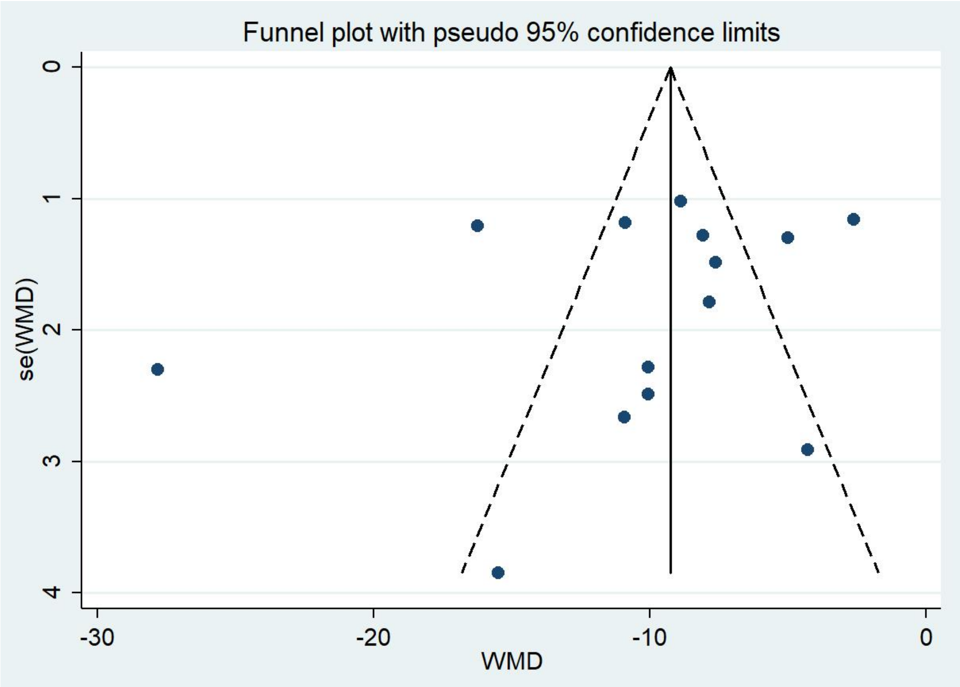

Grade II hypertension - Sensitivity Tests

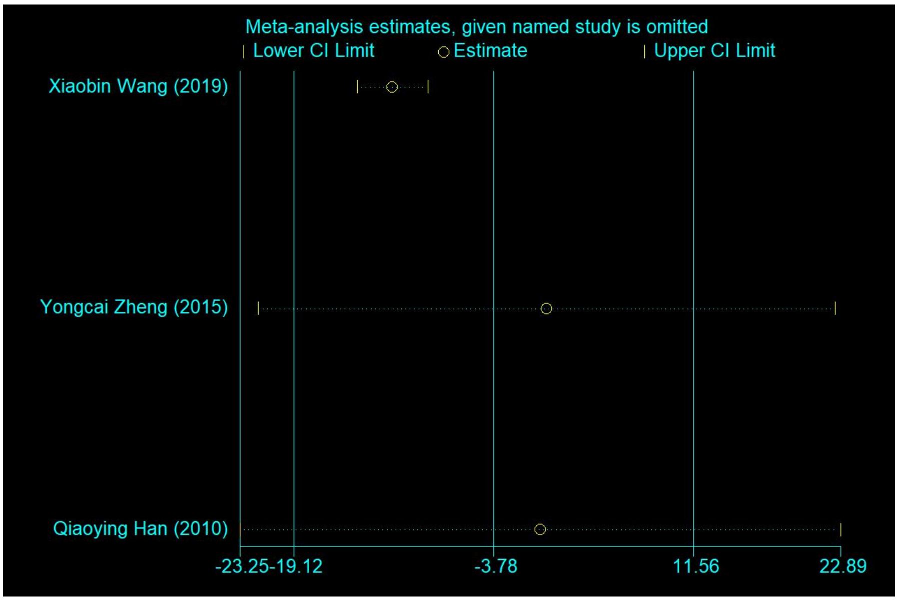

Grade II hypertension - bias testing

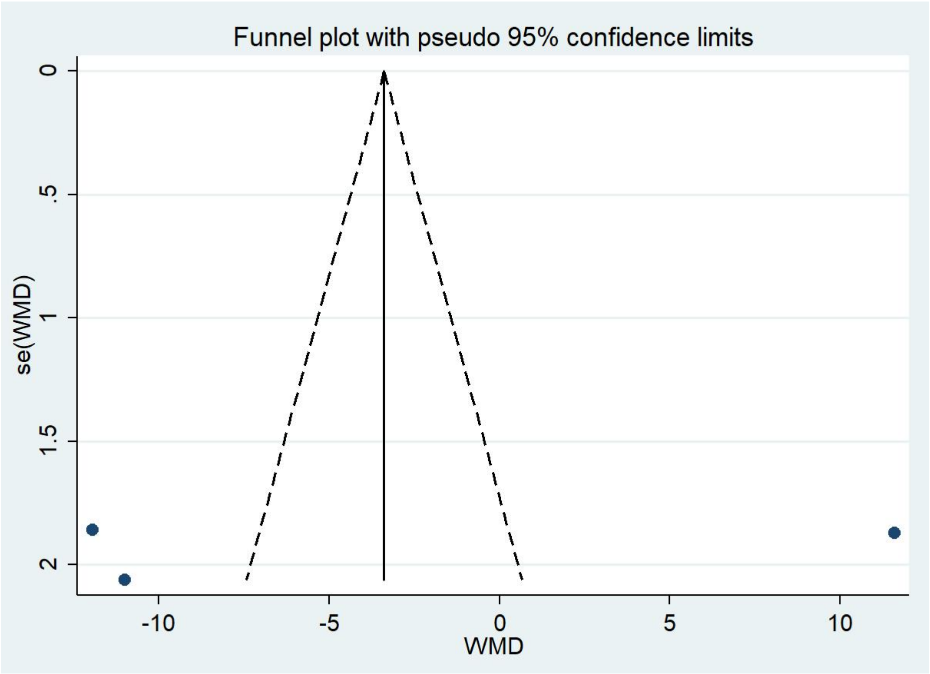

Grade II hypertension -Sensitivity analysis after elimination

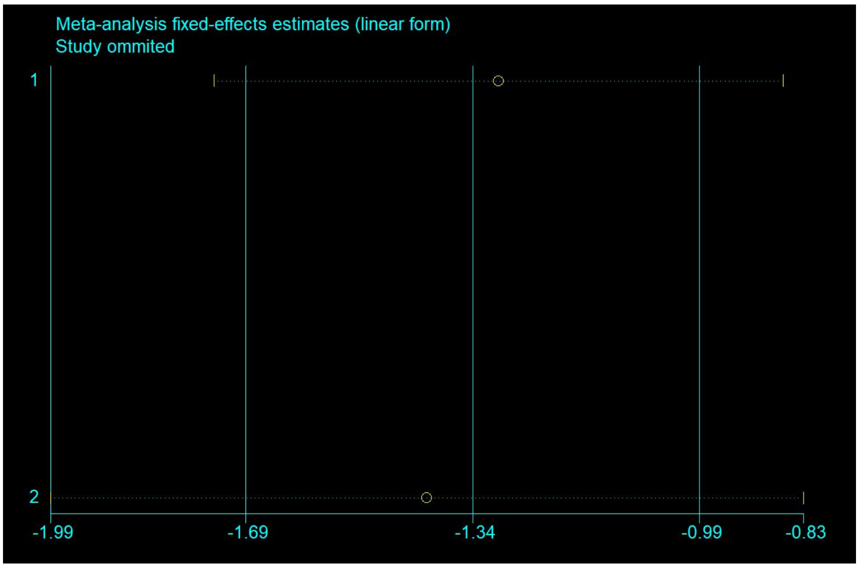

Grade II hypertension - Culled funnel diagram

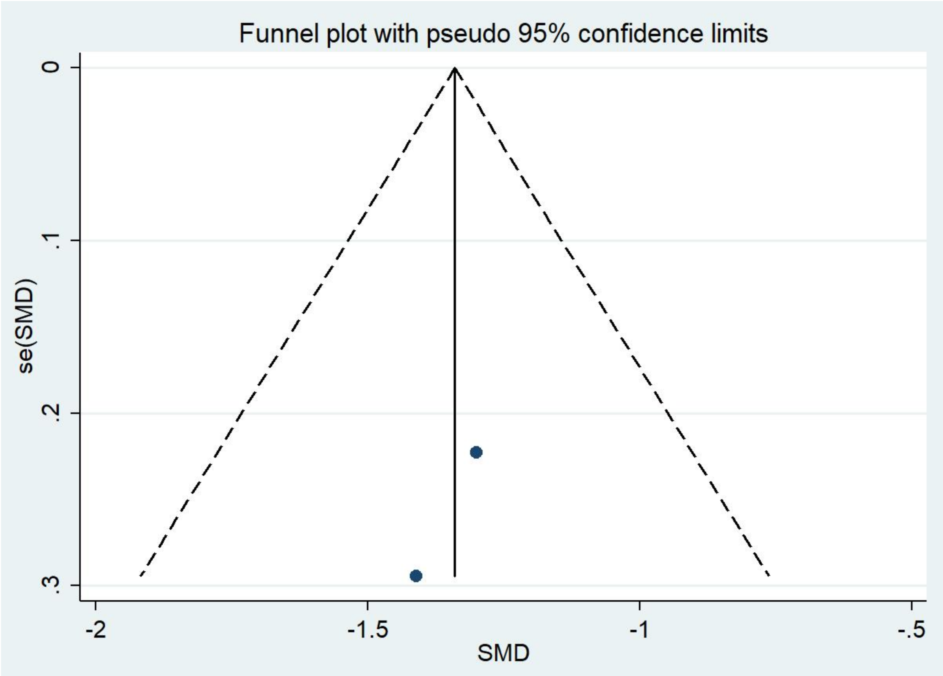

DBP

All - Sensitivity Tests

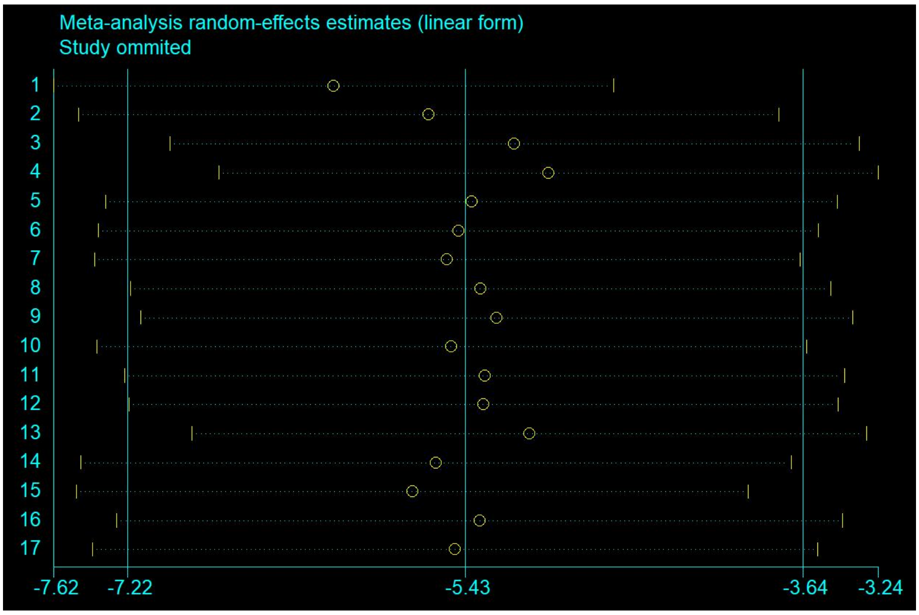

All - bias testing

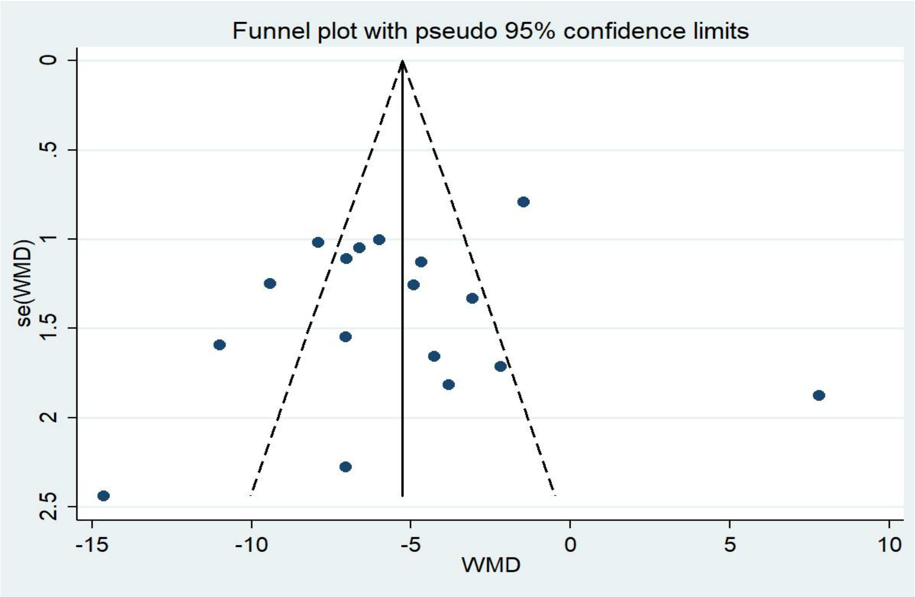

12W - Sensitivity Tests

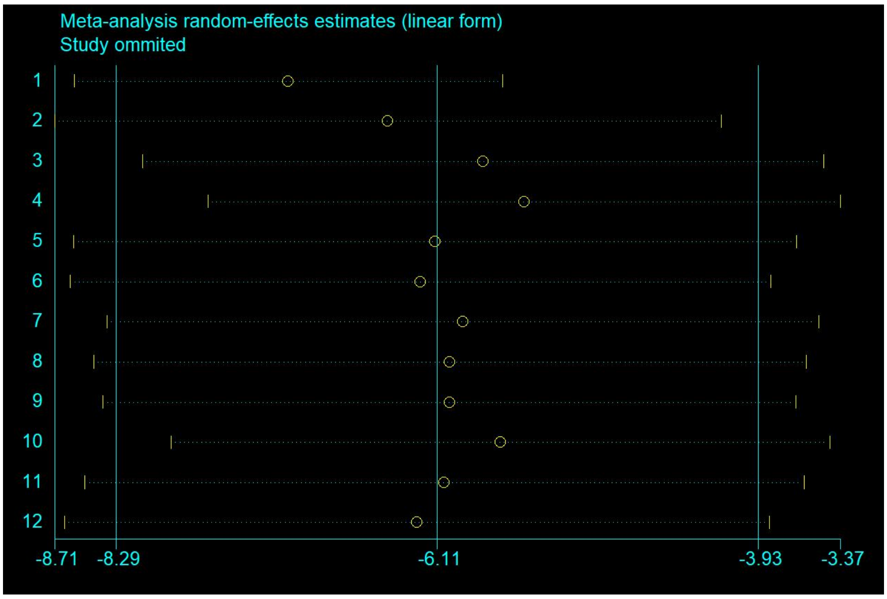

12W - bias testing

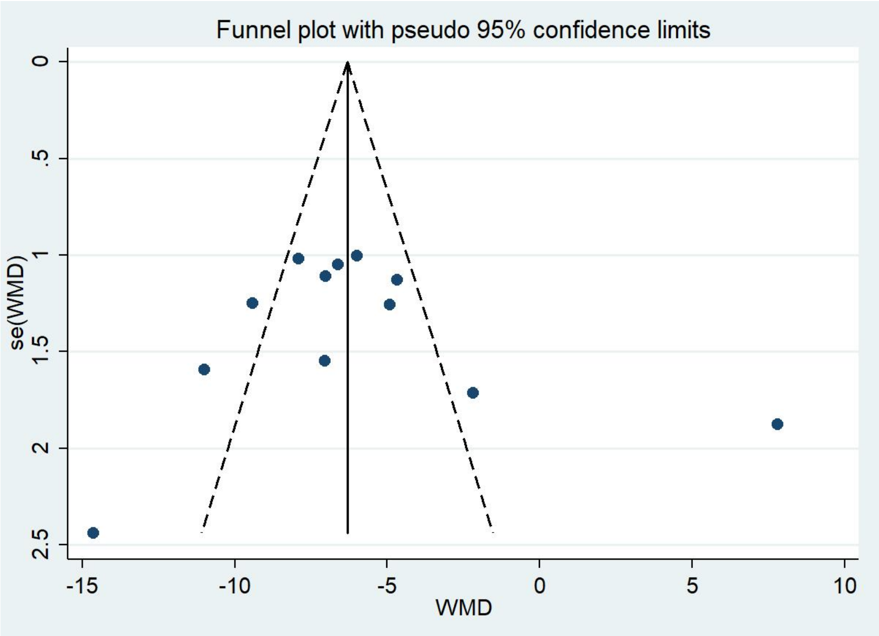

12W or more - Sensitivity Tests

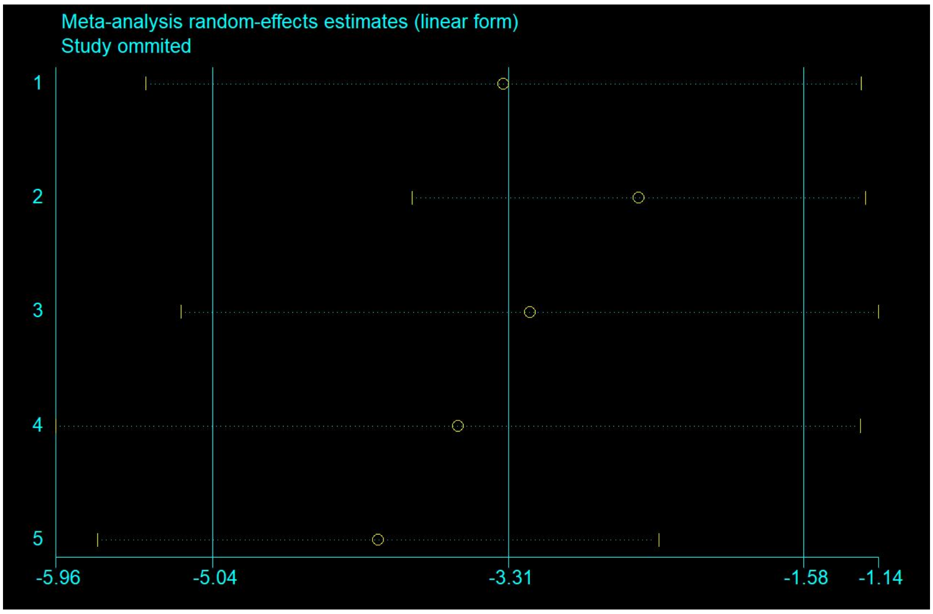

12W or more - bias testing

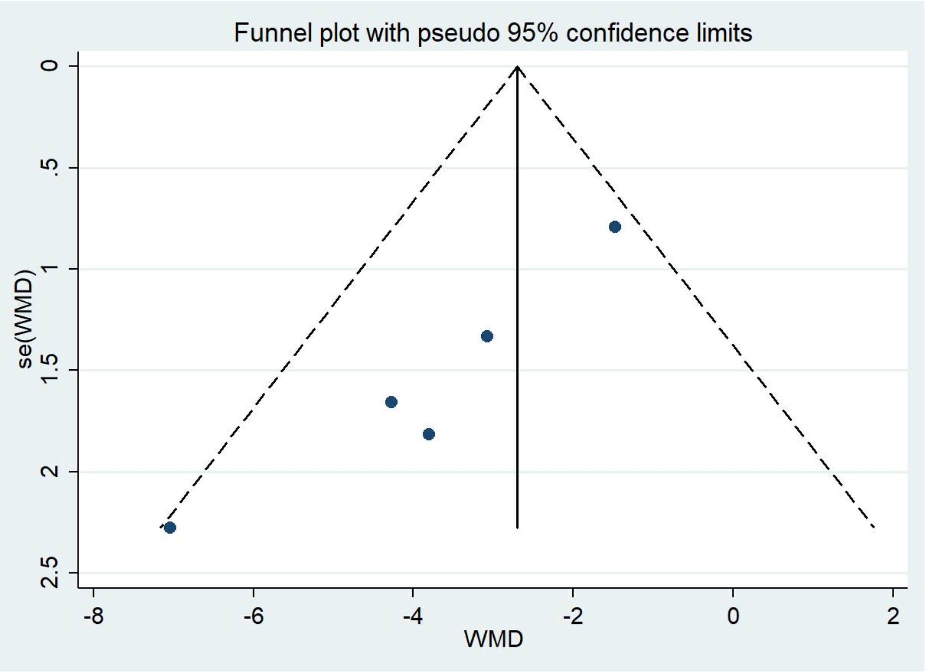

Grade I hypertension - Sensitivity Tests

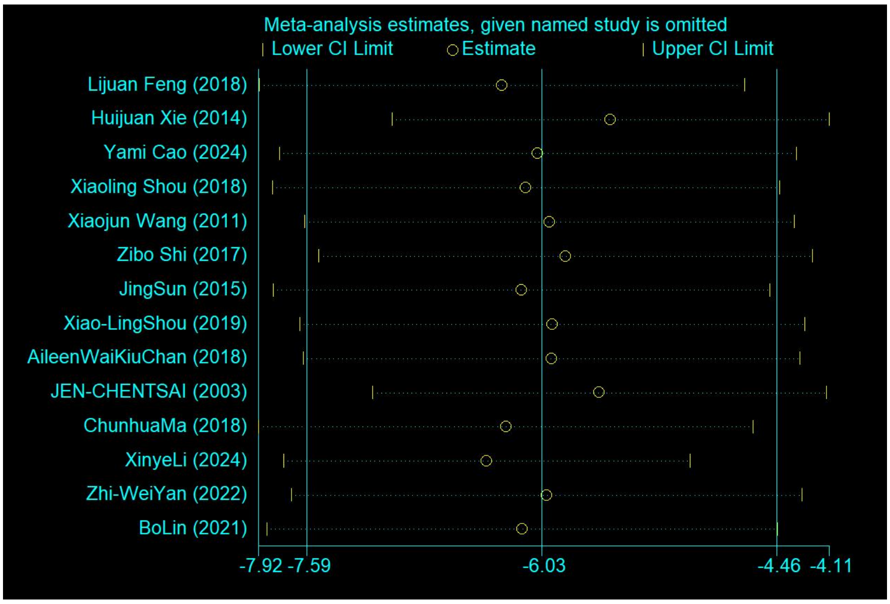

Grade I hypertension - bias testing

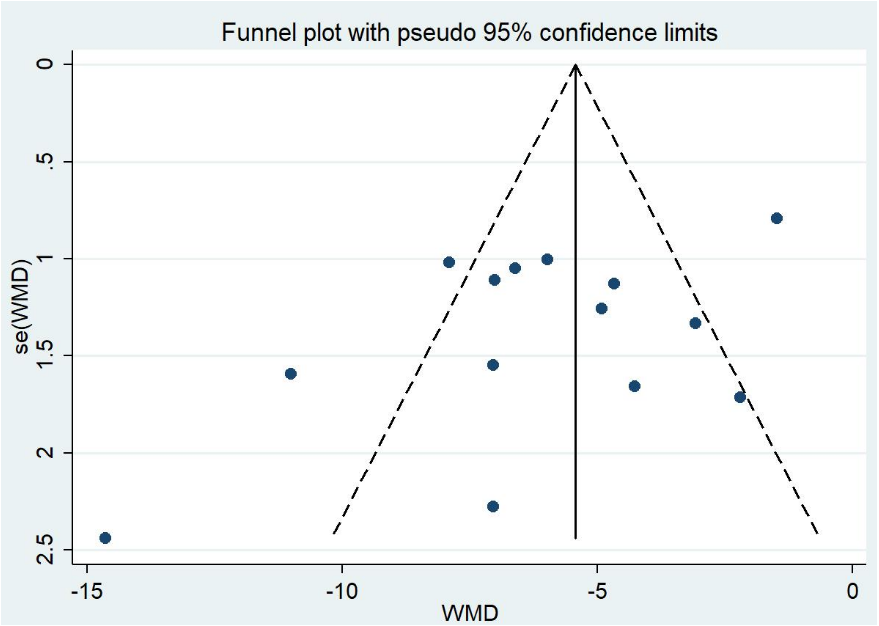

Grade II hypertension - Sensitivity Tests

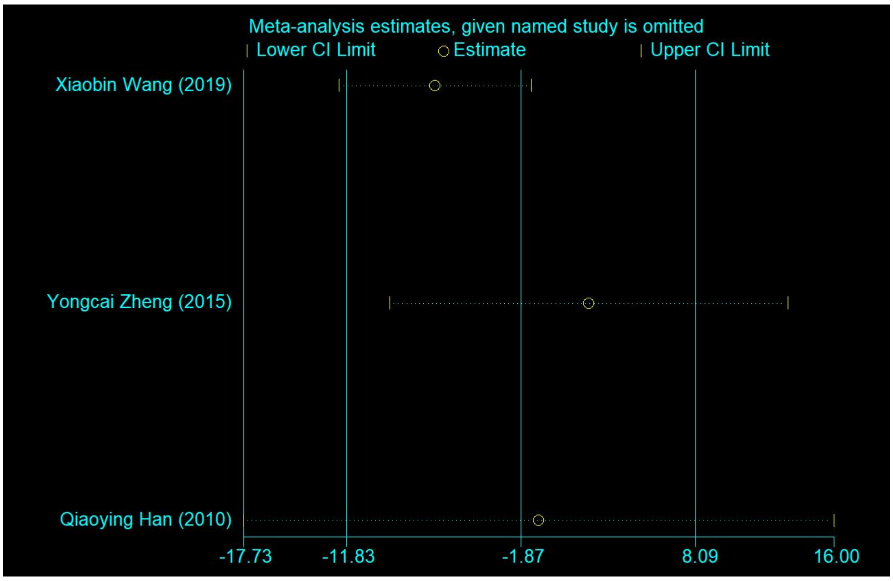

Grade II hypertension - bias testing

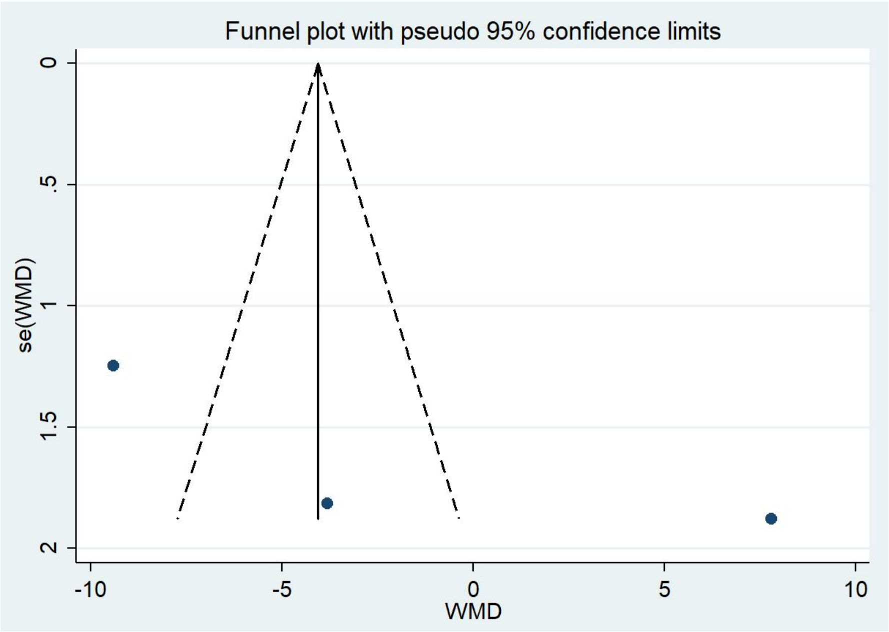

TC

All - Sensitivity Tests

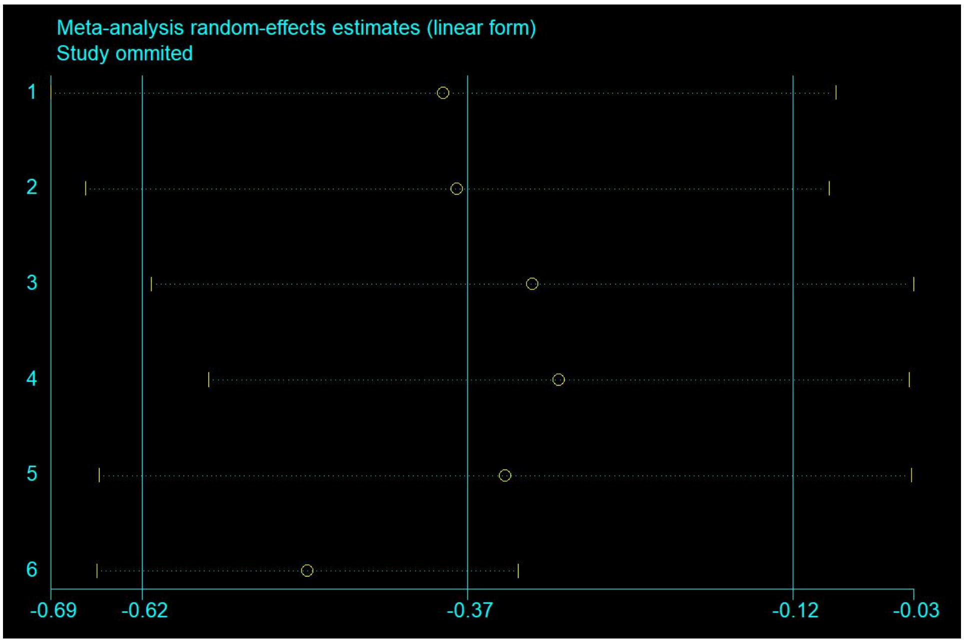

Sensitivity analysis after elimination

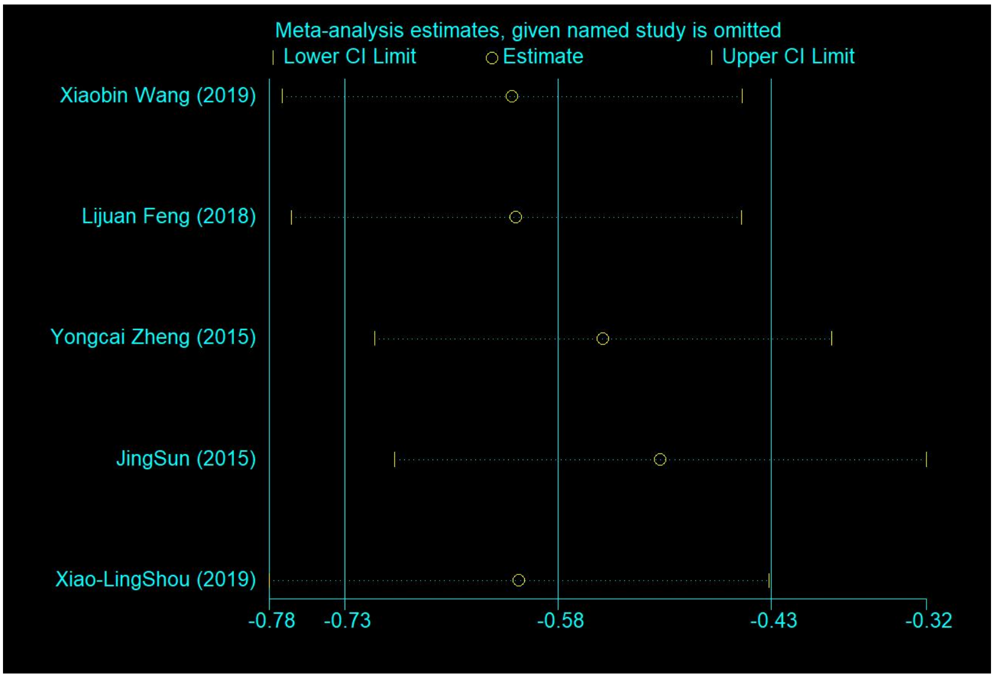

All - bias testing

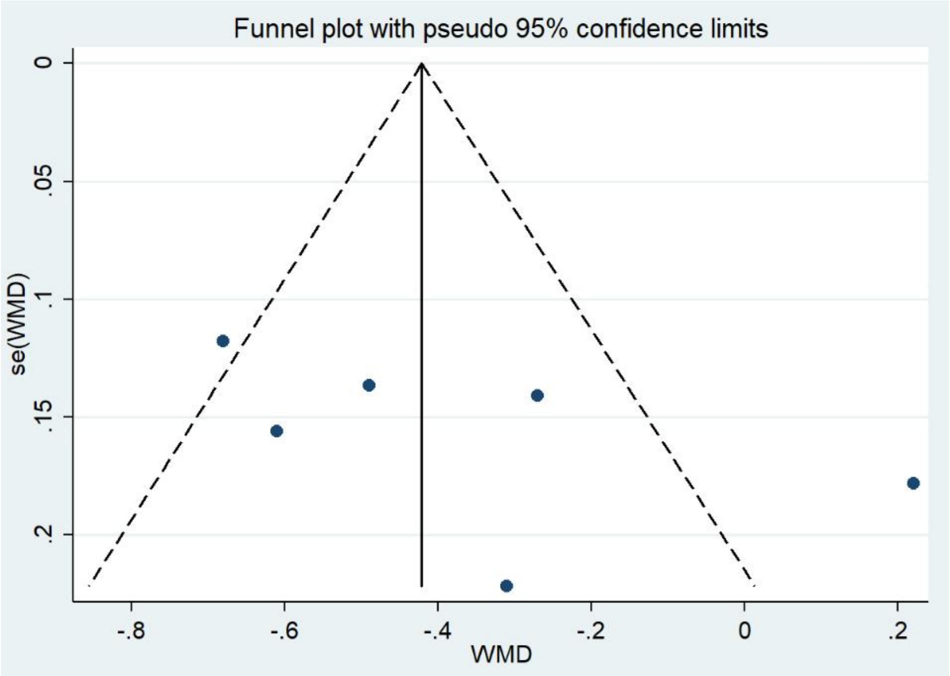

Culled funnel diagram

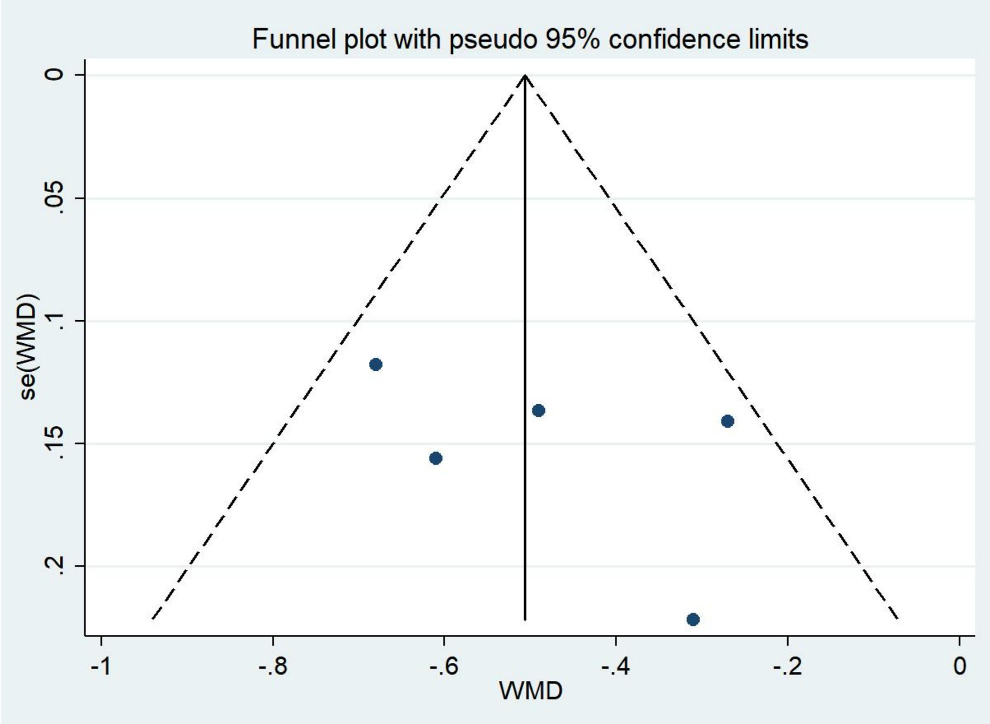

TG

All - Sensitivity Tests

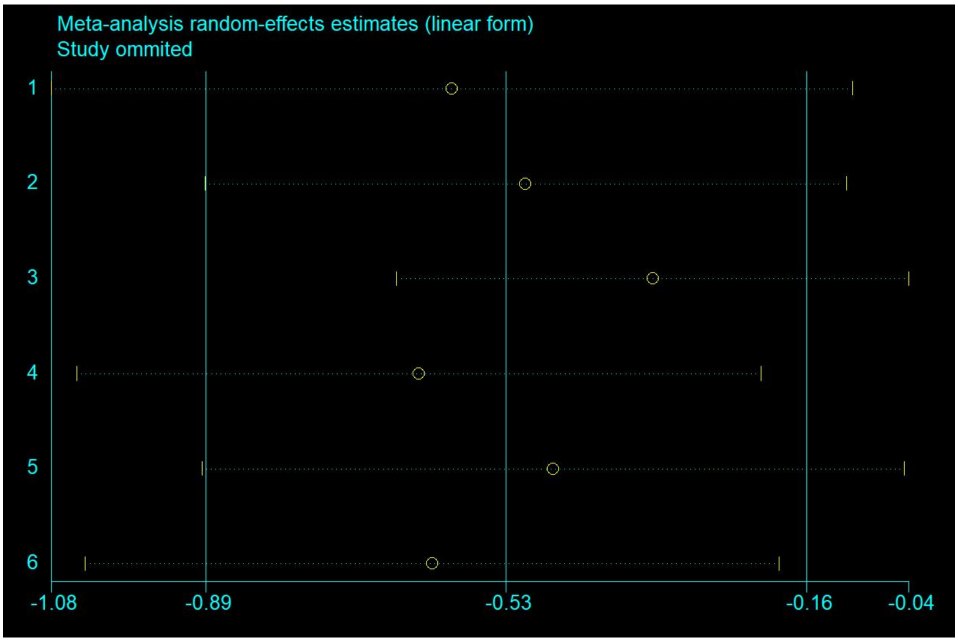

All - bias testing

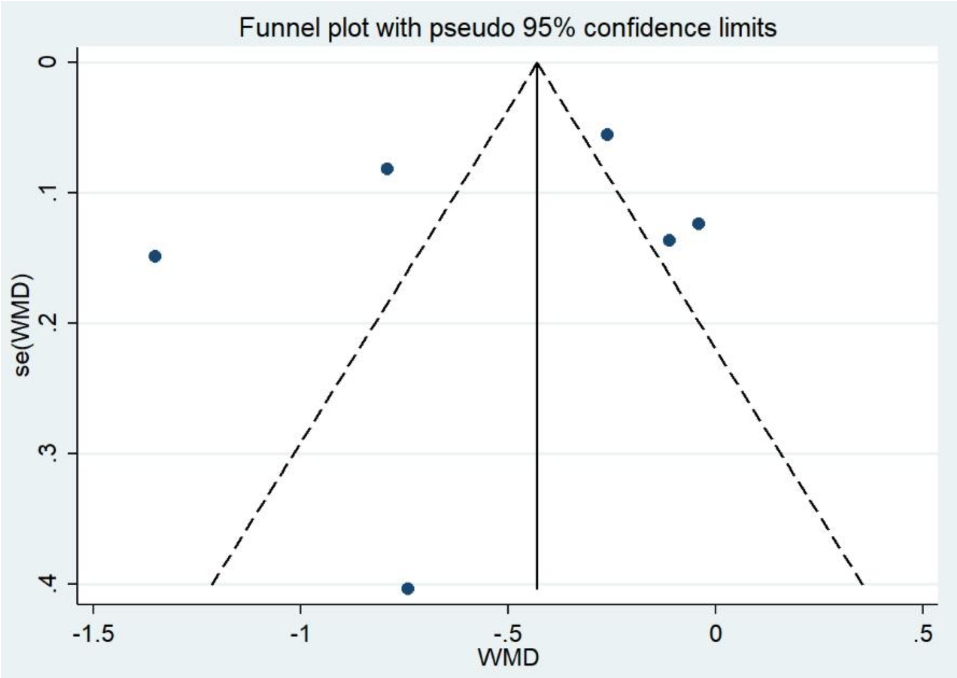

HDL

All - Sensitivity Tests

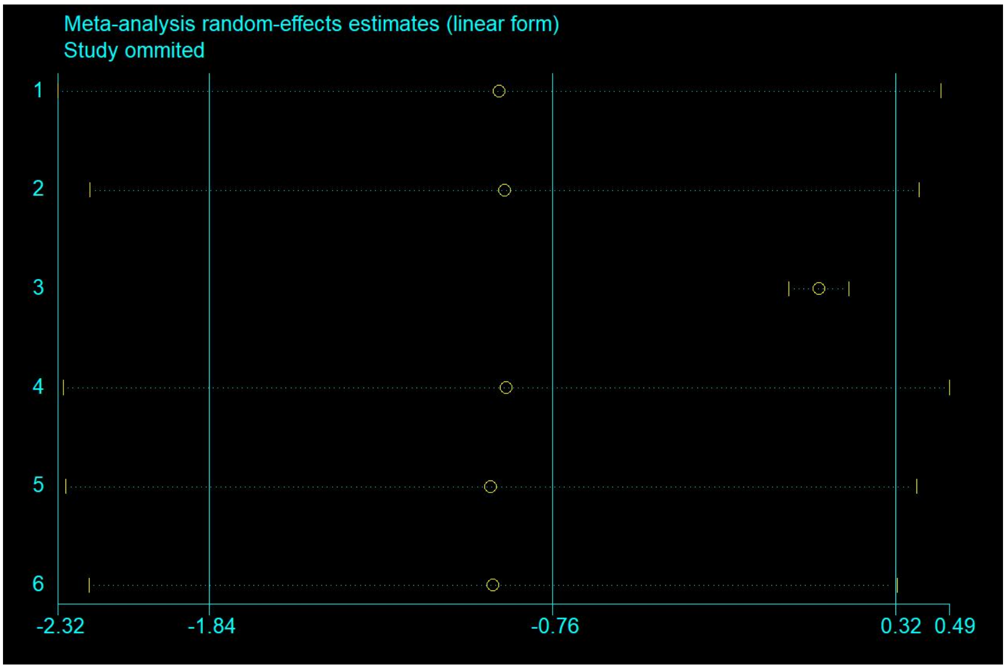

All - bias testing

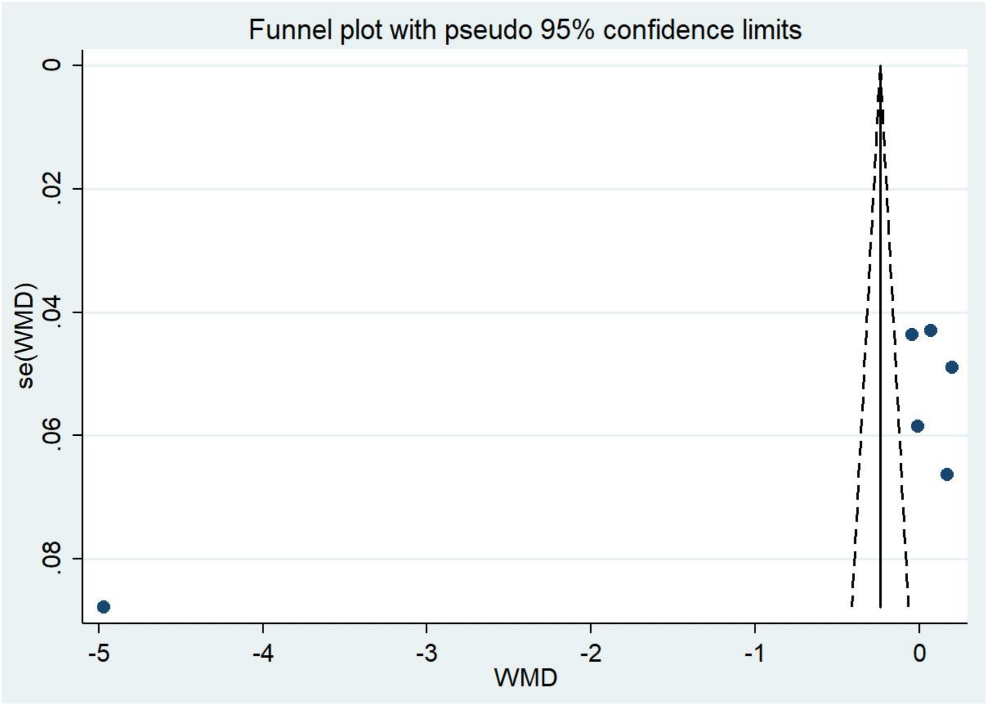

LDL

All - Sensitivity Tests

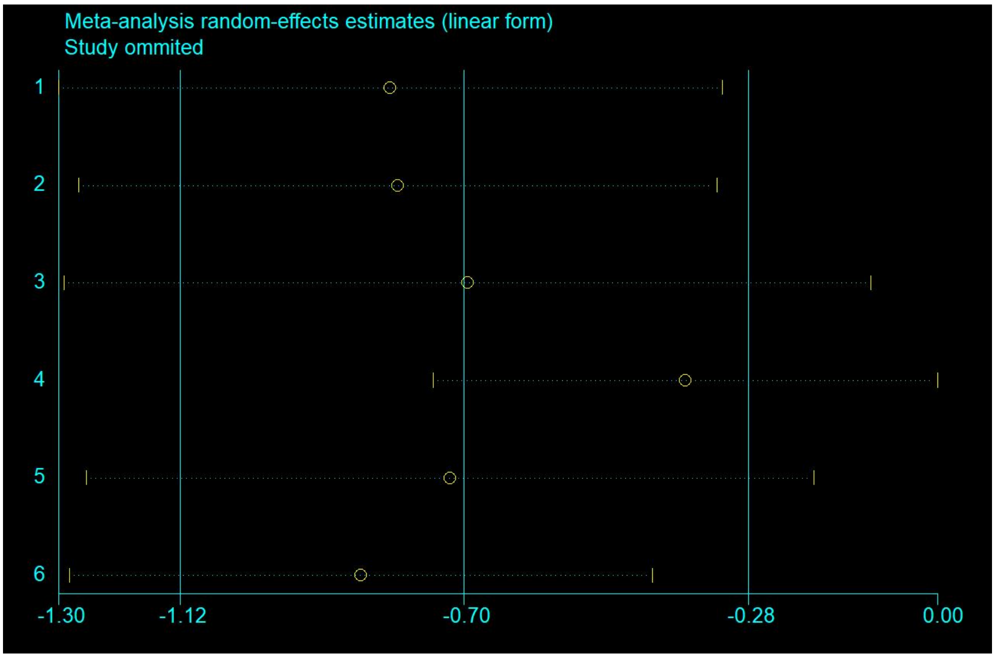

All - bias testing

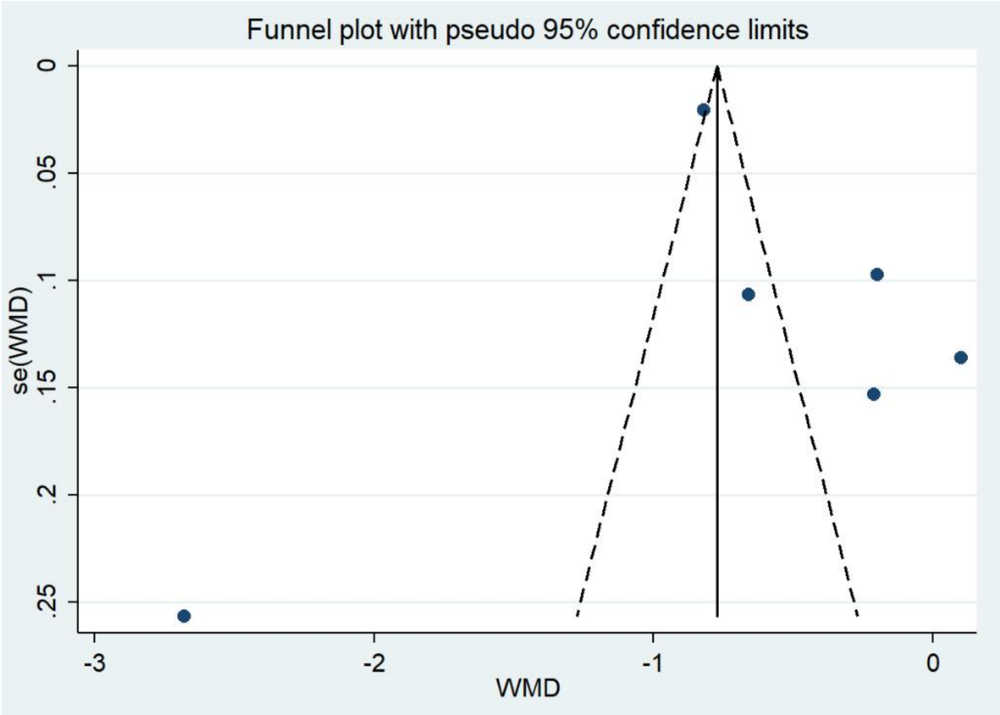

BMI

All - Sensitivity Tests

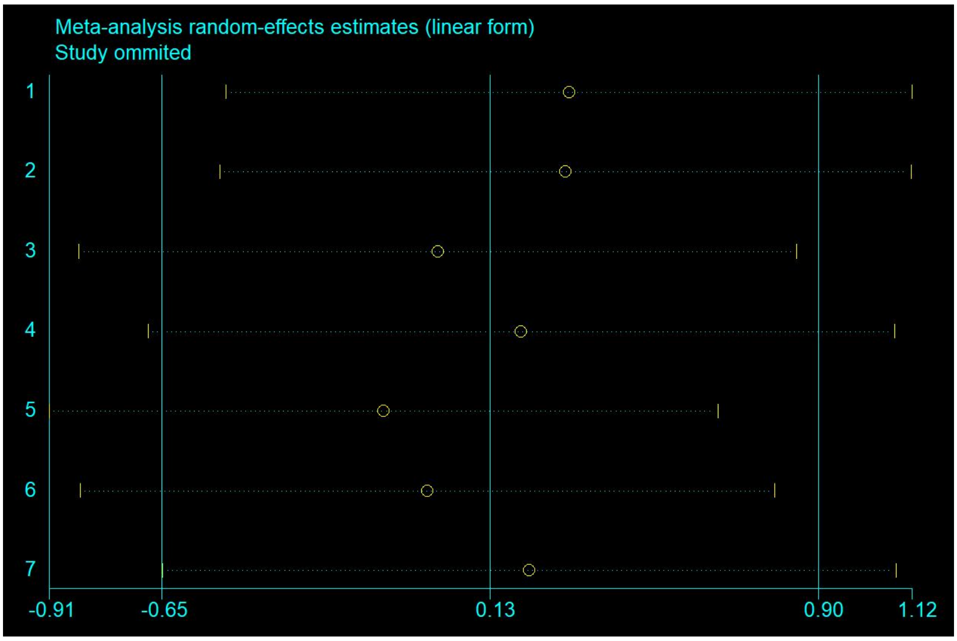

All - bias testing

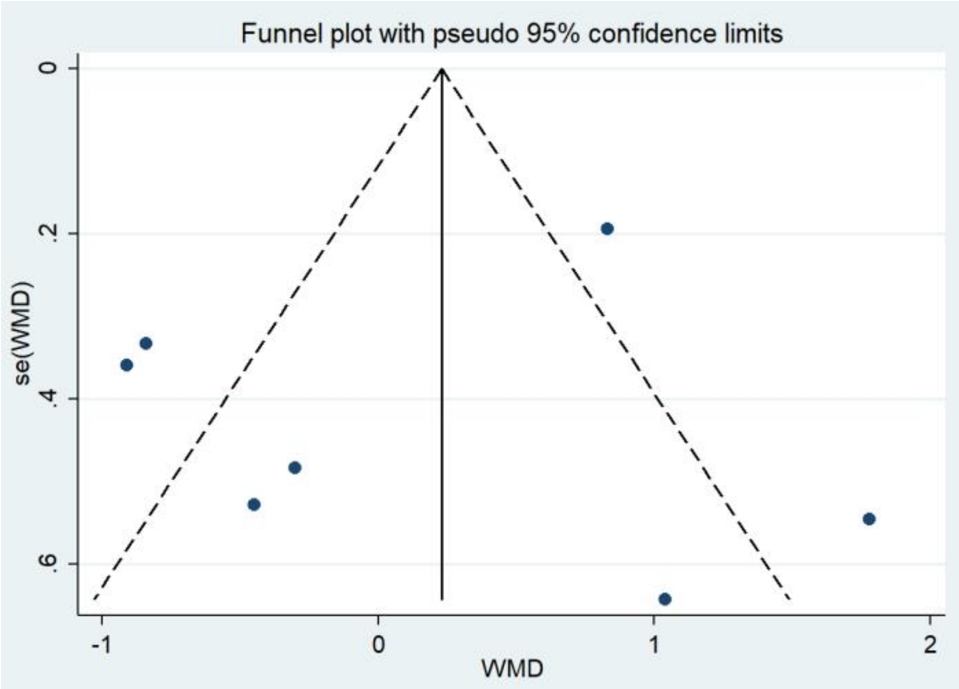

WC

All - Sensitivity Tests

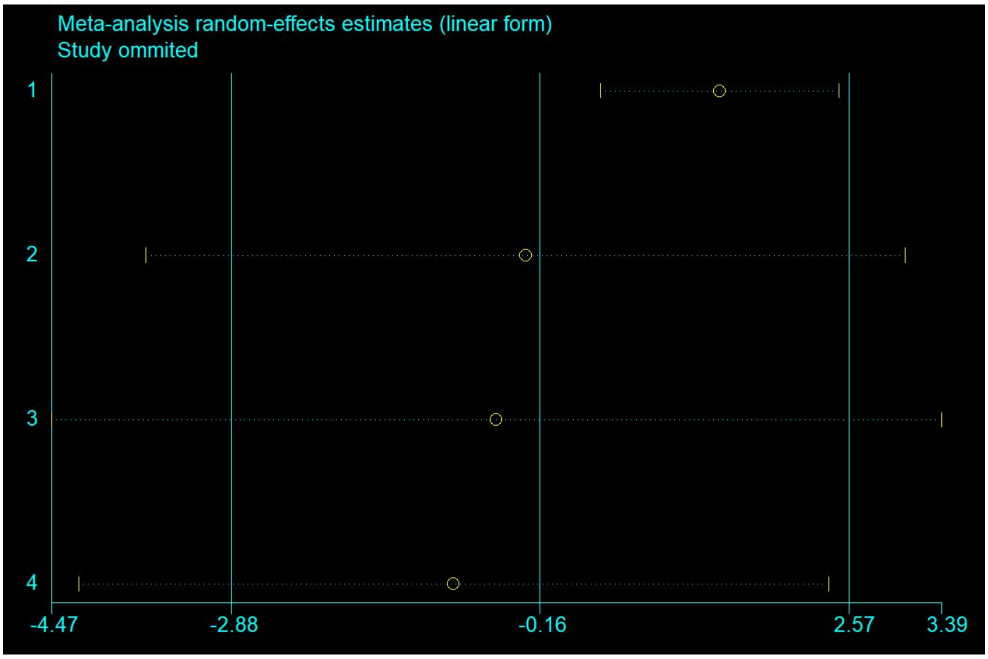

Sensitivity analysis after elimination

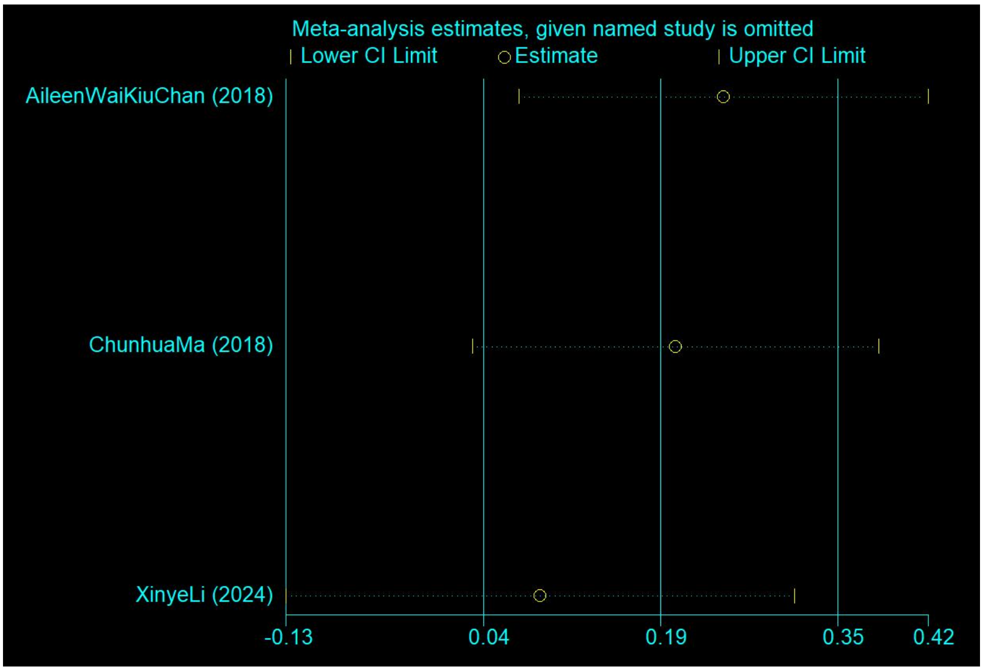

All - bias testing

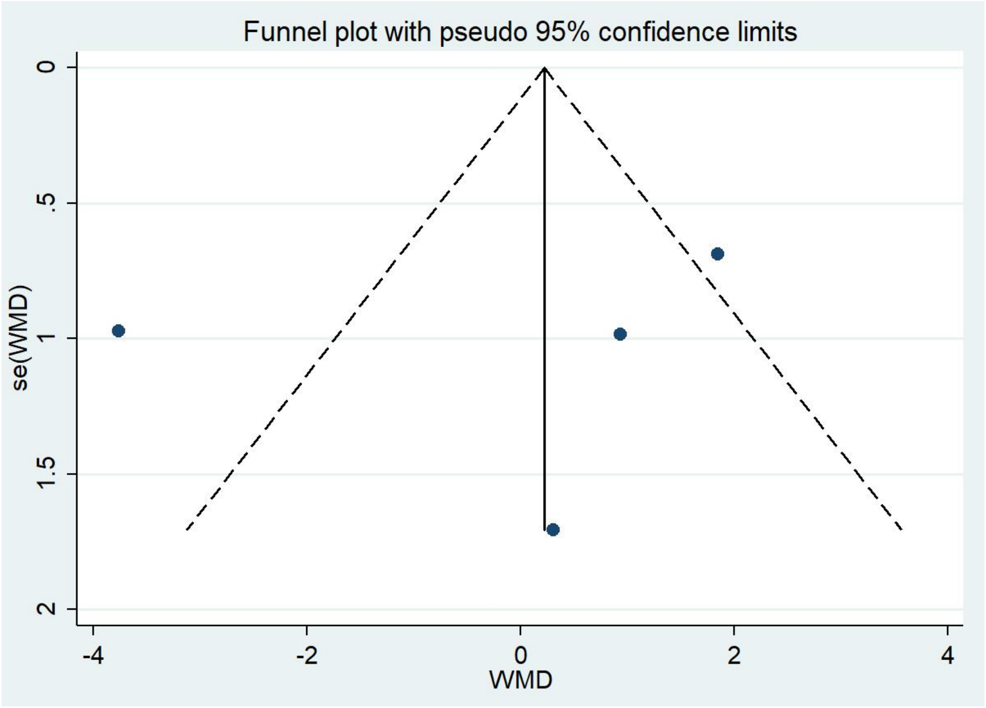

Culled funnel diagram

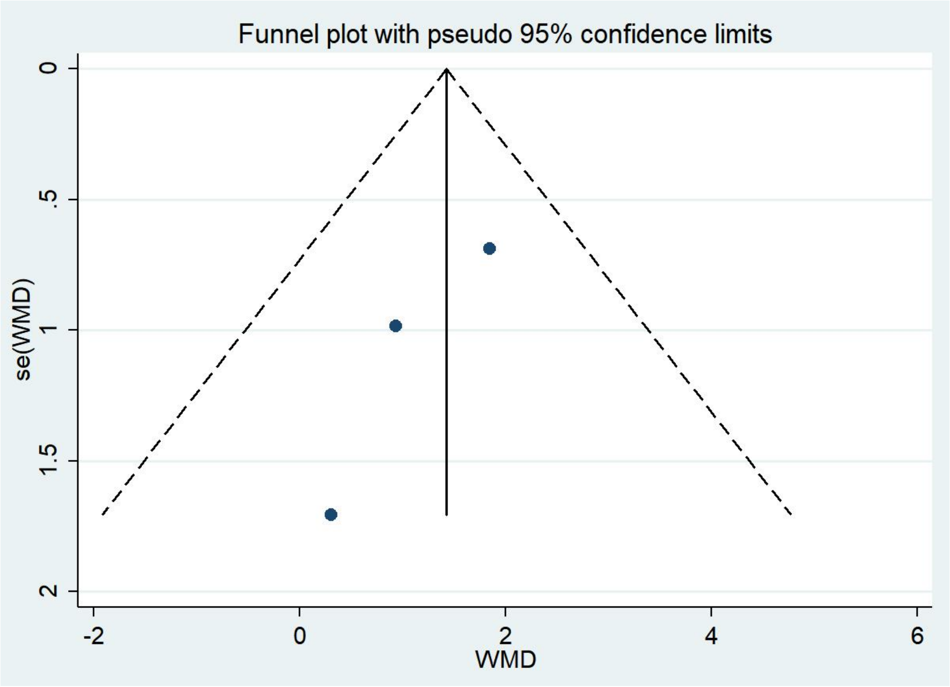

High-quality research sensitivity analyses

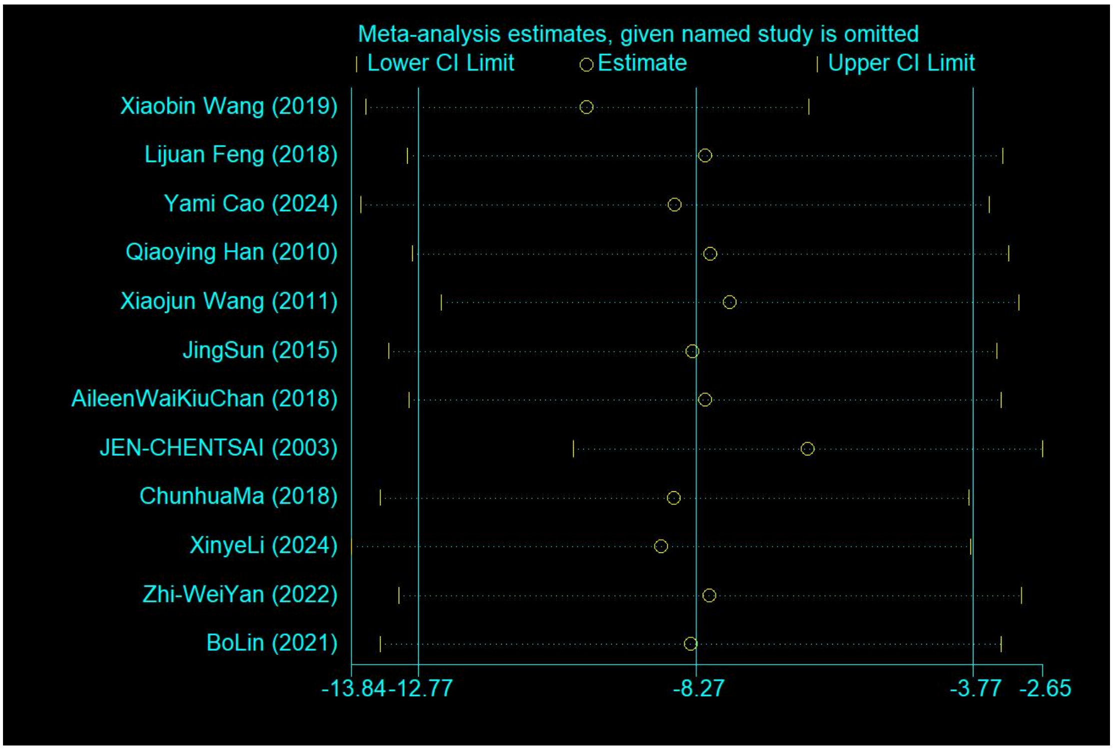

Search strategy

1. Search Format for CNKI, WANFANG and CBM :
#1 (Topic: Taichi) OR (Topic: taijiquan) OR (Topic: taiji) OR (Topic: taichi exercise) OR (Topic: taijiquan sport)
#2 (Topic: Hypertension) OR (Topic: Blood Pressure) OR (Topic: High Blood Pressure)
#3 #1 AND #2
2. Search Format for PubMed:
#1 "Hypertension"[Mesh]
#2 (Blood Pressure*[Title/Abstract]) OR (High Blood Pressure*[Title/Abstract])
#3 #1 OR #2
#4 "Tai Ji"[Mesh]
#5 ((((((tai chi[Title/Abstract]) OR (taiji[Title/Abstract])) OR (tai ji[Title/Abstract])) OR (taji*[Title/Abstract])) OR (taijiquan[Title/Abstract])) OR (Tai Chih[Title/Abstract])) OR (chi tai[Title/Abstract])
#6 #4 OR #5
#7 #3 AND #6
3. Search Format for Embase:
#1 ‘hypertension’/exp OR hypertension
#2 'Blood Pressure*':ab,ti OR 'High Blood Pressure*':ab,ti
#3 #1 OR #2
#4 taiji
#5 'tai chi':ab,ti OR 'taiji':ab,ti OR 'tai ji':ab,ti OR 'taji*':ab,ti OR 'taijiquan':ab,ti OR 'Tai Chih':ab,ti OR 'chi tai':ab,ti
#6 #4 OR #5
#7 #3 AND #6
4. Search Format for The Cochrane Library:
#1 ‘hypertension’
#2 (Blood Pressure*):ab,ti,kw OR (High Blood Pressure*):ab,ti,kw
#3 #1 OR #2
#4 Tai Ji
#5 (tai chi):ab,ti,kw OR (taiji):ab,ti,kw OR (tai ji):ab,ti,kw OR (taji*):ab,ti,kw OR (taijiquan):ab,ti,kw OR (Tai Chih):ab,ti,kw OR (chi tai):ab,ti,kw
#6 #4 OR #5
#7 #3 AND #6

Meta-analysis results graph

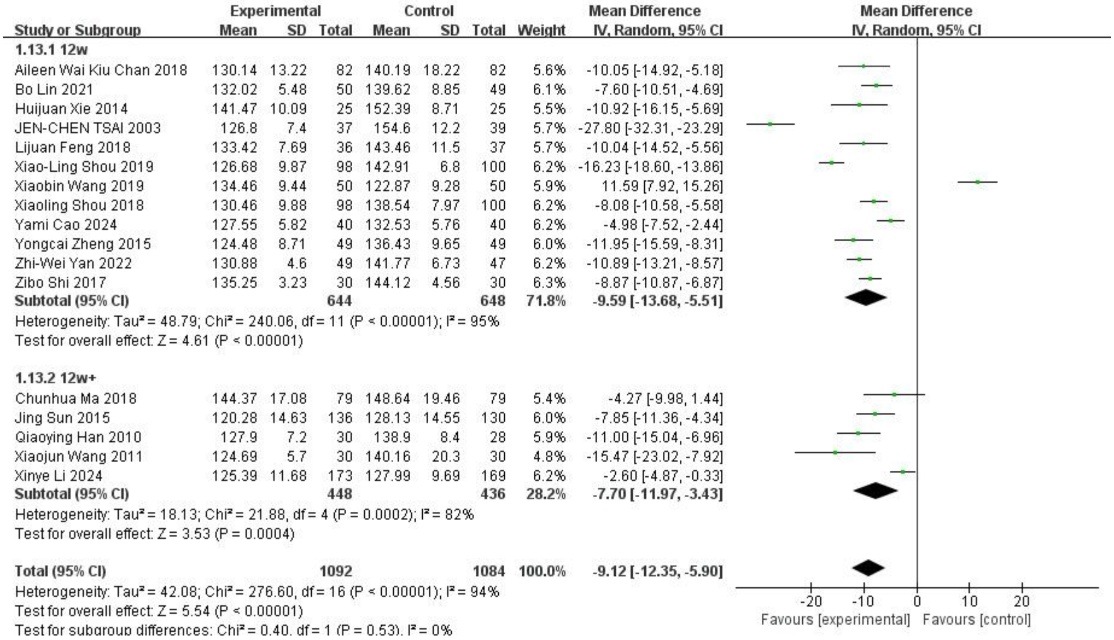

Fig. 4. Effect of tai chi exercise on SBP levels in hypertensive patients (Duration of intervention)

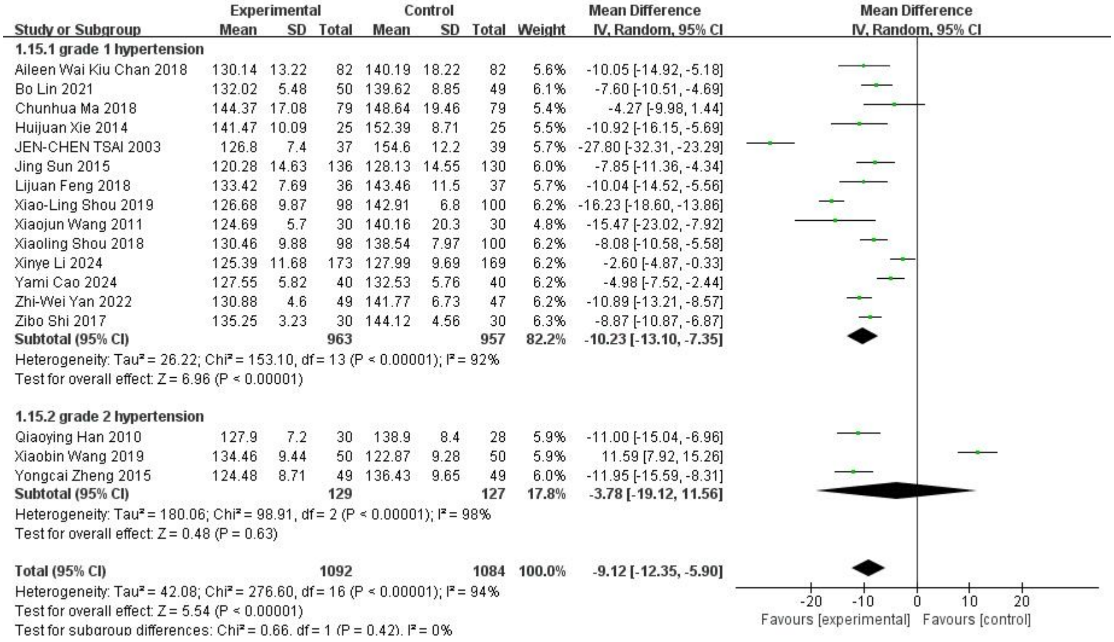

Study or Subgroup	Experimental Mean	Experimental SD	Experimental Total	Control Mean	Control SD	Control Total	Weight	Mean Difference IV, Random, 95% CI
**1.15.1 grade 1 hypertension**								
Aileen Wai Kiu Chan 2018	130.14	13.22	82	140.19	18.22	82	5.8%	-10.05 [-14.92, -5.18]
Bo Lin 2021	132.02	5.48	50	139.62	8.85	49	6.7%	-7.60 [-10.51, -4.69]
Chunhua Ma 2018	144.37	17.08	79	148.64	19.46	79	5.4%	-4.27 [-9.98, 1.44]
Huijuan Xie 2014	141.47	10.09	25	152.39	8.71	25	5.6%	-10.92 [-16.15, -5.69]
JEN-CHEN TSAI 2003	126.8	7.4	37	154.6	12.2	39	6.0%	-27.80 [-32.31, -23.29]
Jing Sun 2015	120.28	14.63	136	128.13	14.55	130	6.4%	-7.85 [-11.36, -4.34]
Lijuan Feng 2018	133.42	7.69	36	143.46	11.5	37	6.0%	-10.04 [-14.52, -5.56]
Xiao-Ling Shou 2019	126.68	9.87	98	142.91	6.8	100	6.9%	-16.23 [-18.60, -13.86]
Xiaojun Wang 2011	124.69	5.7	30	140.16	20.3	30	4.5%	-15.47 [-23.02, -7.92]
Xiaoling Shou 2018	130.46	9.88	98	138.54	7.97	100	6.8%	-8.08 [-10.58, -5.58]
Xinye Li 2024	125.39	11.68	173	127.99	9.69	169	6.9%	-2.60 [-4.87, -0.33]
Yami Cao 2024	127.55	5.82	40	132.53	5.76	40	6.8%	-4.98 [-7.52, -2.44]
Zhi-Wei Yan 2022	130.88	4.6	49	141.77	6.73	47	6.9%	-10.89 [-13.21, -8.57]
Zibo Shi 2017	135.25	3.23	30	144.12	4.56	30	7.0%	-8.87 [-10.87, -6.87]
**Subtotal (95% CI)**			**963**			**957**	**87.5%**	**-10.23 [-13.10, -7.35]**
Heterogeneity: Tau² = 26.22; Chi² = 153.10, df = 13 (P < 0.00001); I² = 92%								
Test for overall effect: Z = 6.96 (P < 0.00001)								
**1.15.2 grade 2 hypertension**								
Qiaoying Han 2010	127.9	7.2	30	138.9	8.4	28	6.2%	-11.00 [-15.04, -6.96]
Xiaobin Wang 2019	134.46	9.44	50	122.87	9.28	50	0.0%	11.59 [7.92, 15.26]
Yongcai Zheng 2015	124.48	8.71	49	136.43	9.65	49	6.4%	-11.95 [-15.59, -8.31]
**Subtotal (95% CI)**			**79**			**77**	**12.5%**	**-11.52 [-14.23, -8.82]**
Heterogeneity: Tau² = 0.00; Chi² = 0.12, df = 1 (P = 0.73); I² = 0%								
Test for overall effect: Z = 8.35 (P < 0.00001)								
**Total (95% CI)**			**1042**			**1034**	**100.0%**	**-10.37 [-12.95, -7.79]**
Heterogeneity: Tau² = 23.86; Chi² = 155.70, df = 15 (P < 0.00001); I² = 90%								
Test for overall effect: Z = 7.87 (P < 0.00001)								
Test for subgroup differences: Chi² = 0.42, df = 1 (P = 0.52), I² = 0%								

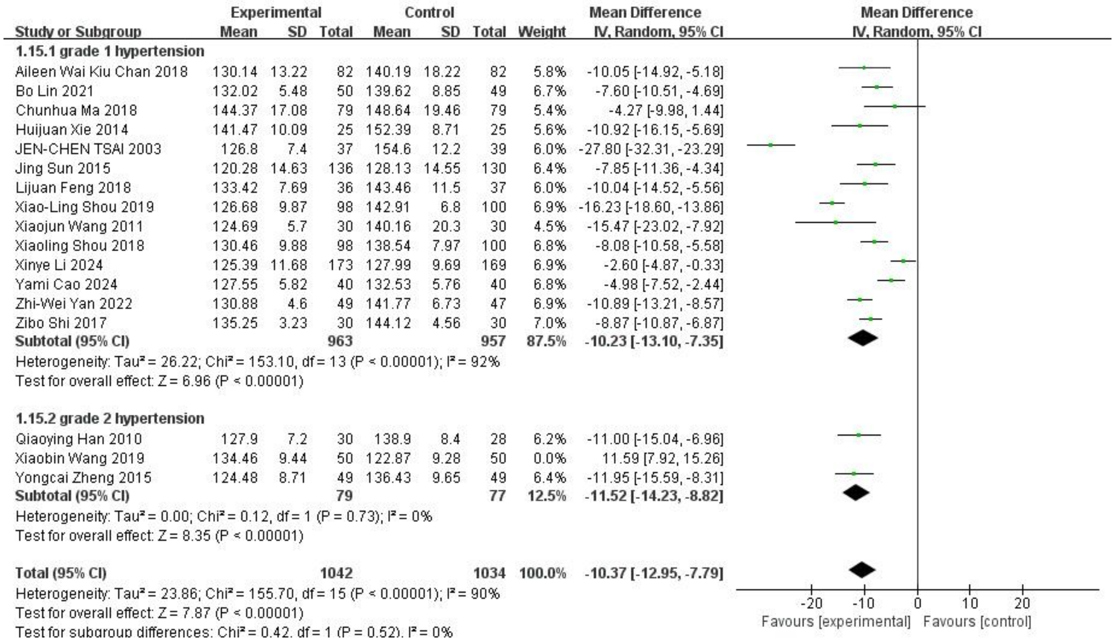

Fig. 5. Effect of tai chi exercise on SBP levels in hypertensive patients (Grade)

Study or Subgroup	Experimental Mean	Experimental SD	Experimental Total	Control Mean	Control SD	Control Total	Weight	Mean Difference IV, Random, 95% CI
**1.14.1 12w**								
Aileen Wai Kiu Chan 2018	76.78	9.61	82	83.82	10.19	82	5.8%	-7.04 [-10.07, -4.01]
Bo Lin 2021	76.43	6.68	50	81.1	4.31	49	6.3%	-4.67 [-6.88, -2.46]
Huijuan Xie 2014	83.59	9.67	25	98.23	7.43	25	4.7%	-14.64 [-19.42, -9.86]
JEN-CHEN TSAI 2003	78.6	6	37	89.6	7.8	39	5.7%	-11.00 [-14.12, -7.88]
Lijuan Feng 2018	78.86	7.32	36	81.05	7.33	37	5.6%	-2.19 [-5.55, 1.17]
Xiao-Ling Shou 2019	76.28	7.79	98	83.29	7.8	100	6.3%	-7.01 [-9.18, -4.84]
Xiaobin Wang 2019	76.24	10.23	50	68.44	8.46	50	5.4%	7.80 [4.12, 11.48]
Xiaoling Shou 2018	82.29	7.82	98	87.2	9.77	100	6.2%	-4.91 [-7.37, -2.45]
Yami Cao 2024	78.63	4.5	40	84.61	4.48	40	6.4%	-5.98 [-7.95, -4.01]
Yongcai Zheng 2015	75.32	3.84	49	84.73	7.84	49	6.2%	-9.41 [-11.85, -6.97]
Zhi-Wei Yan 2022	79.39	5.25	49	86	5.04	47	6.4%	-6.61 [-8.67, -4.55]
Zibo Shi 2017	81.58	4.31	30	89.48	3.53	30	6.4%	-7.90 [-9.89, -5.91]
**Subtotal (95% CI)**			**644**			**648**	**71.3%**	**-6.11 [-8.29, -3.93]**
Heterogeneity: Tau² = 12.76; Chi² = 95.40, df = 11 (P < 0.00001); I² = 88%								
Test for overall effect: Z = 5.50 (P < 0.00001)								
**1.14.2 12w+**								
Chunhua Ma 2018	84.53	8.91	79	87.6	7.78	79	6.1%	-3.07 [-5.68, -0.46]
Jing Sun 2015	75.31	14.53	136	79.58	12.44	130	5.7%	-4.27 [-7.52, -1.02]
Qiaoying Han 2010	83.4	6.7	30	87.2	7.1	28	5.5%	-3.80 [-7.36, -0.24]
Xiaojun Wang 2011	77.34	5.92	30	84.38	10.98	30	4.9%	-7.04 [-11.50, -2.58]
Xinye Li 2024	80.47	6.98	173	81.94	7.67	169	6.6%	-1.47 [-3.03, 0.09]
**Subtotal (95% CI)**			**448**			**436**	**28.7%**	**-3.31 [-5.04, -1.58]**
Heterogeneity: Tau² = 1.70; Chi² = 7.38, df = 4 (P = 0.12); I² = 46%								
Test for overall effect: Z = 3.75 (P = 0.0002)								
**Total (95% CI)**			**1092**			**1084**	**100.0%**	**-5.43 [-7.22, -3.64]**
Heterogeneity: Tau² = 12.02; Chi² = 130.68, df = 16 (P < 0.00001); I² = 88%								
Test for overall effect: Z = 5.94 (P < 0.00001)								
Test for subgroup differences: Chi² = 3.90, df = 1 (P = 0.05), I² = 74.3%								

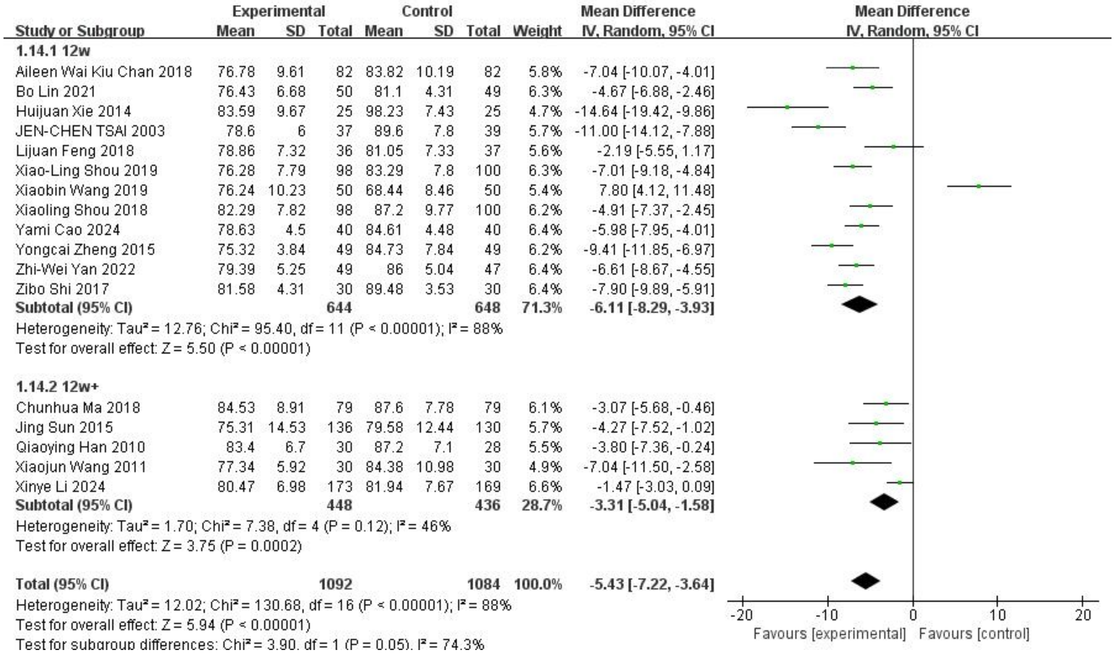

Fig. 6. Effect of tai chi exercise on DBP levels in hypertensive patients (Duration of intervention)

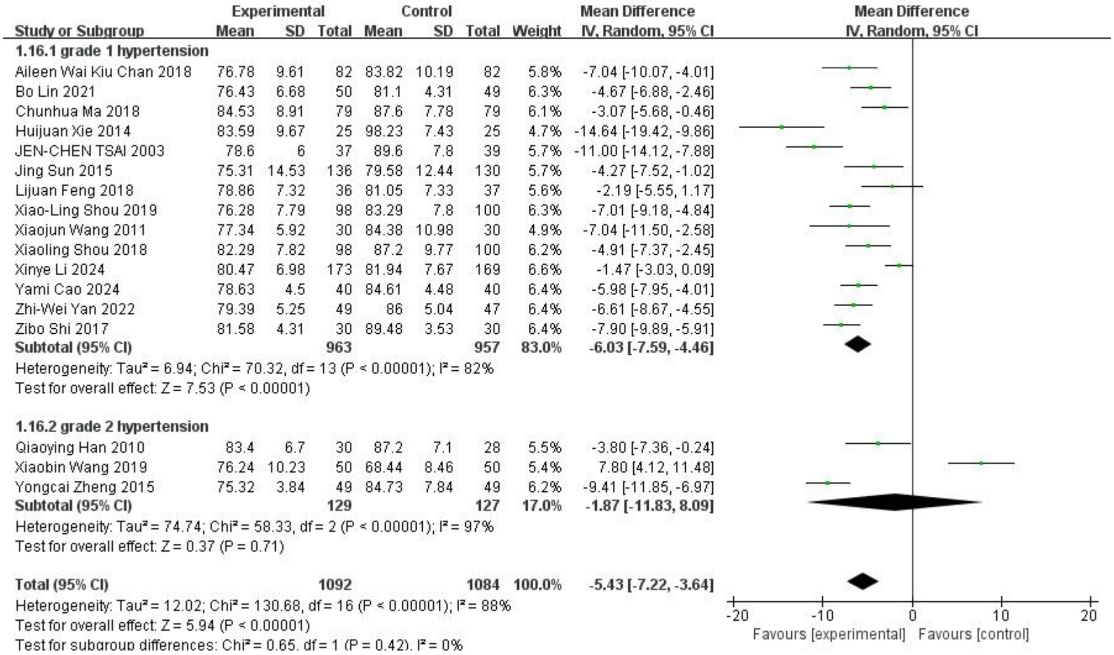

Fig. 7. Effect of tai chi exercise on DBP levels in hypertensive patients (Grade)

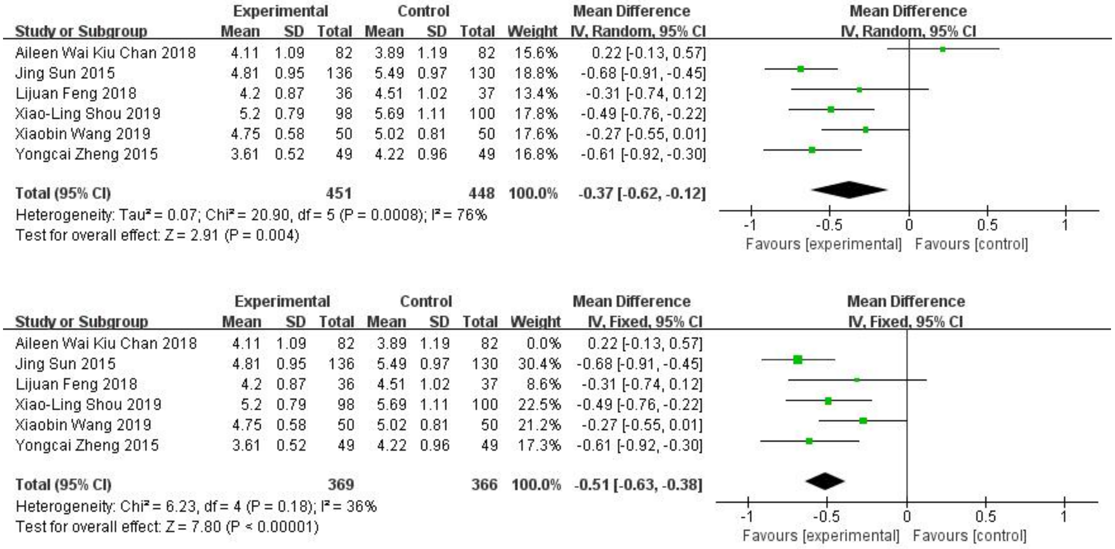

Fig. 8. Forest plot comparing TC in Tai Chi and all controls.

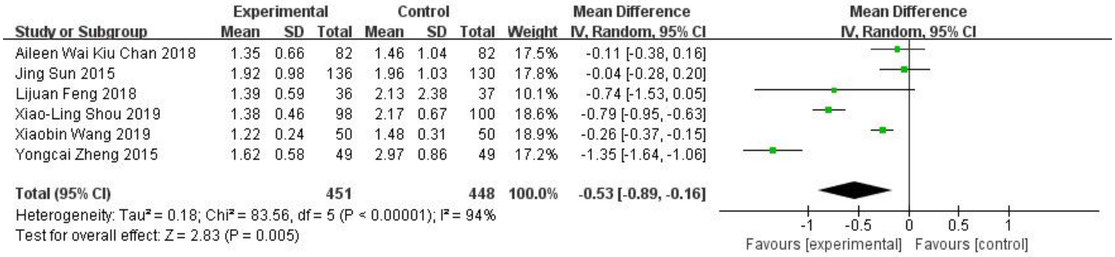

Fig. 9. Forest plot comparing TG in TC and all controls.

Study or Subgroup	Experimental Mean	Experimental SD	Experimental Total	Control Mean	Control SD	Control Total	Weight	Mean Difference IV, Random, 95% CI
Aileen Wai Kiu Chan 2018	2.39	0.78	82	2.29	0.95	82	16.8%	0.10 [-0.17, 0.37]
Jing Sun 2015	2.73	0.79	136	5.41	2.82	130	14.3%	-2.68 [-3.18, -2.18]
Lijuan Feng 2018	2.12	0.58	36	2.33	0.72	37	16.5%	-0.21 [-0.51, 0.09]
Xiao-Ling Shou 2019	2.6	0.49	98	3.26	0.94	100	17.2%	-0.66 [-0.87, -0.45]
Xiaobin Wang 2019	3.1	0.5	50	3.3	0.47	50	17.3%	-0.20 [-0.39, -0.01]
Yongcai Zheng 2015	1.69	0.06	49	2.51	0.13	49	17.9%	-0.82 [-0.86, -0.78]
**Total (95% CI)**			**451**			**448**	**100.0%**	**-0.70 [-1.12, -0.28]**

Heterogeneity: Tau² = 0.26; Chi² = 151.51, df = 5 (P < 0.00001); I² = 97%
Test for overall effect: Z = 3.24 (P = 0.001)

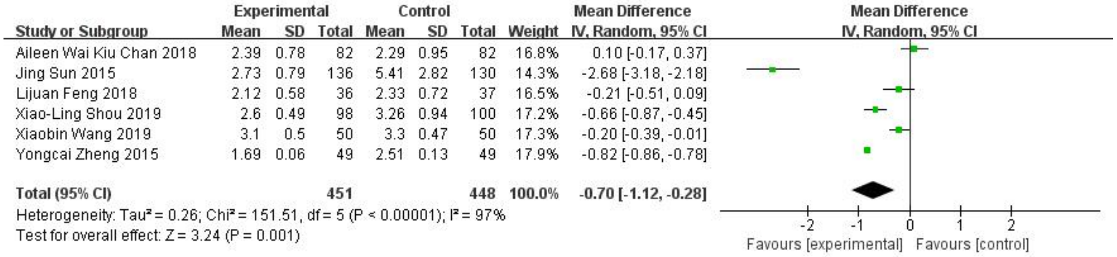

Fig. 10. Forest plot comparing LDL in TC and all controls.

Study or Subgroup	Experimental Mean	Experimental SD	Experimental Total	Control Mean	Control SD	Control Total	Weight	Mean Difference IV, Random, 95% CI
Aileen Wai Kiu Chan 2018	1.28	0.43	82	1.11	0.42	82	16.7%	0.17 [0.04, 0.30]
Jing Sun 2015	1.59	0.34	136	1.63	0.37	130	16.7%	-0.04 [-0.13, 0.05]
Lijuan Feng 2018	1.29	0.24	36	1.3	0.26	37	16.7%	-0.01 [-0.12, 0.10]
Xiao-Ling Shou 2019	1.49	0.25	98	1.29	0.42	100	16.7%	0.20 [0.10, 0.30]
Xiaobin Wang 2019	1.32	0.21	50	1.25	0.22	50	16.7%	0.07 [-0.01, 0.15]
Yongcai Zheng 2015	1.35	0.08	49	6.32	0.61	49	16.6%	-4.97 [-5.14, -4.80]
**Total (95% CI)**			**451**			**448**	**100.0%**	**-0.76 [-1.84, 0.32]**

Heterogeneity: Tau² = 1.81; Chi² = 3103.51, df = 5 (P < 0.00001); I² = 100%
Test for overall effect: Z = 1.38 (P = 0.17)

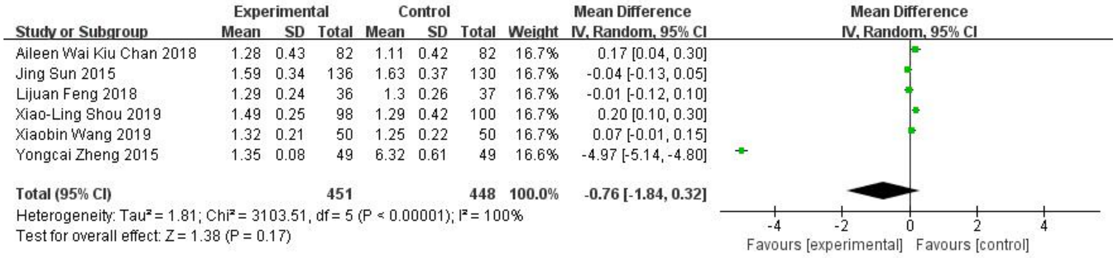

Fig. 11. Forest plot comparing HDL in TC and all controls.

Study or Subgroup	Experimental Mean	Experimental SD	Experimental Total	Control Mean	Control SD	Control Total	Weight	Mean Difference IV, Random, 95% CI
Aileen Wai Kiu Chan 2018	26.09	4.33	82	25.05	3.89	82	12.0%	1.04 [-0.22, 2.30]
Chunhua Ma 2018	24.23	3.02	79	22.45	3.79	79	13.1%	1.78 [0.71, 2.85]
JEN-CHEN TSAI 2003	23.6	2.2	37	23.9	2	39	13.9%	-0.30 [-1.25, 0.65]
Jing Sun 2015	22.25	2.91	136	23.16	2.94	130	15.3%	-0.91 [-1.61, -0.21]
Xiao-Ling Shou 2019	25.83	2.71	98	26.67	1.9	100	15.6%	-0.84 [-1.49, -0.19]
Xinye Li 2024	25.32	1.57	173	24.49	1.98	169	16.8%	0.83 [0.45, 1.21]
Zhi-Wei Yan 2022	23.74	2.72	49	24.19	2.45	47	13.3%	-0.45 [-1.48, 0.58]
**Total (95% CI)**			**654**			**646**	**100.0%**	**0.13 [-0.65, 0.90]**

Heterogeneity: Tau² = 0.88; Chi² = 42.55, df = 6 (P < 0.00001); I² = 86%
Test for overall effect: Z = 0.32 (P = 0.75)

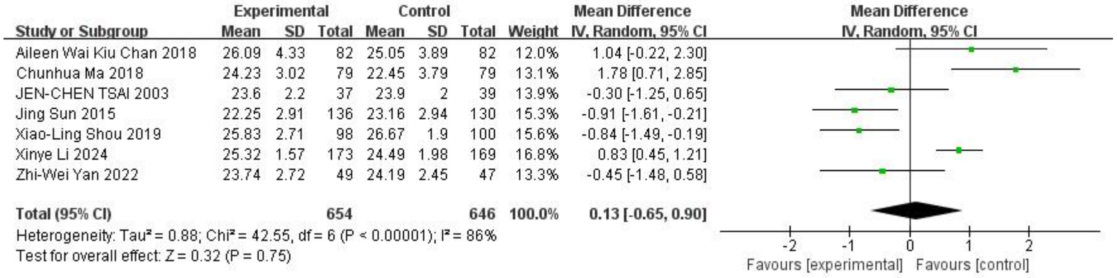

Fig. 12. Forest plot comparing BMI in TC and all controls.

Study or Subgroup	Experimental Mean	Experimental SD	Experimental Total	Control Mean	Control SD	Control Total	Weight	Mean Difference IV, Random, 95% CI
Aileen Wai Kiu Chan 2018	89.85	10.58	82	89.54	11.27	82	20.5%	0.31 [-3.04, 3.66]
Chunhua Ma 2018	74.18	6.37	79	73.24	5.98	79	25.9%	0.94 [-0.99, 2.87]
Jing Sun 2015	75.73	7.56	136	79.49	8.27	130	25.9%	-3.76 [-5.67, -1.85]
Xinye Li 2024	87.31	5.55	173	85.46	7.08	169	27.7%	1.85 [0.50, 3.20]
**Total (95% CI)**			**470**			**460**	**100.0%**	**-0.16 [-2.88, 2.57]**

Heterogeneity: Tau² = 6.50; Chi² = 22.88, df = 3 (P < 0.0001); I² = 87%
Test for overall effect: Z = 0.11 (P = 0.91)

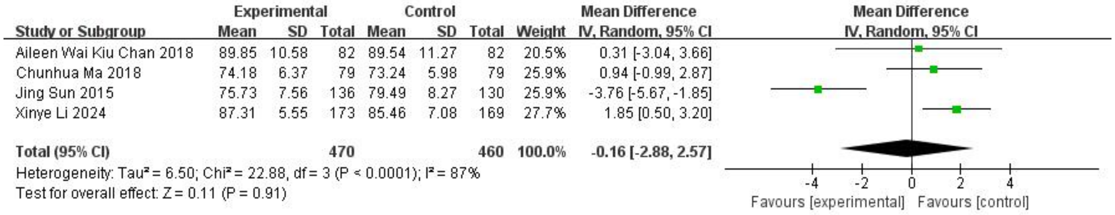

Study or Subgroup	Experimental Mean	Experimental SD	Experimental Total	Control Mean	Control SD	Control Total	Weight	Mean Difference IV, Random, 95% CI
Aileen Wai Kiu Chan 2018	89.85	10.58	82	89.54	11.27	82	9.8%	0.31 [-3.04, 3.66]
Chunhua Ma 2018	74.18	6.37	79	73.24	5.98	79	29.7%	0.94 [-0.99, 2.87]
Jing Sun 2015	75.73	7.56	136	79.49	8.27	130	0.0%	-3.76 [-5.67, -1.85]
Xinye Li 2024	87.31	5.55	173	85.46	7.08	169	60.5%	1.85 [0.50, 3.20]
**Total (95% CI)**			**334**			**330**	**100.0%**	**1.43 [0.38, 2.48]**

Heterogeneity: Tau² = 0.00; Chi² = 1.05, df = 2 (P = 0.59); I² = 0%
Test for overall effect: Z = 2.67 (P = 0.008)

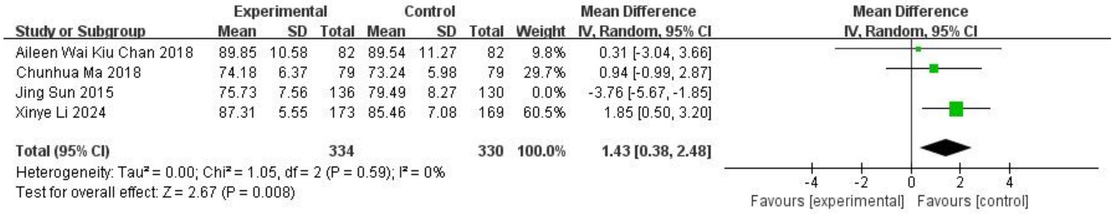

Fig. 13. Forest plot comparing WC in TC and all controls.

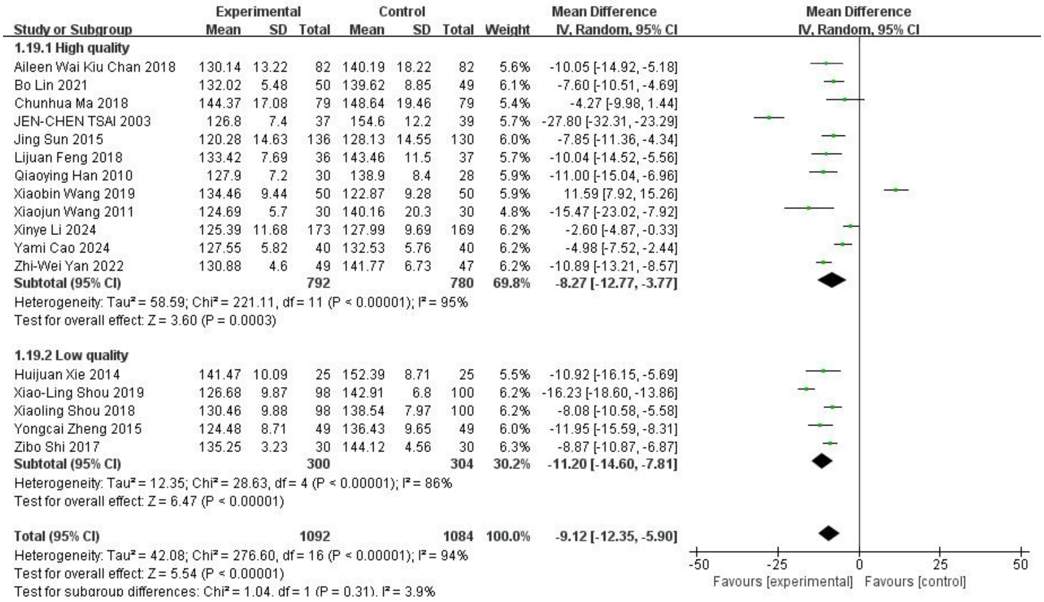

Fig. 14. Forest map of effects of different document quality on SBP

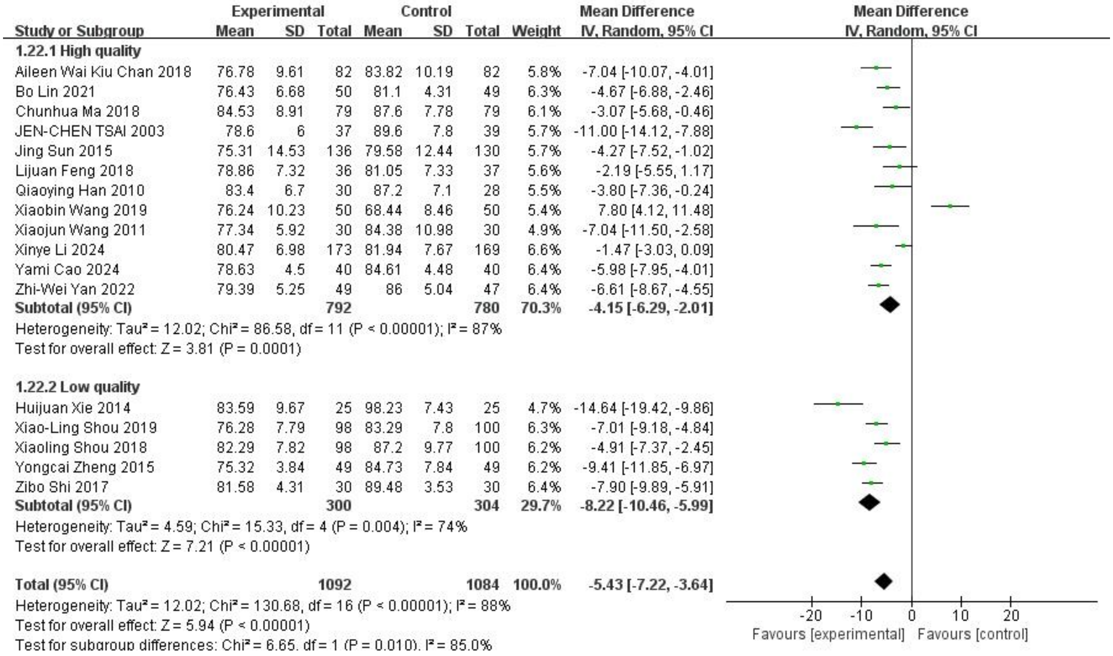

Fig. 15. Forest map of effects of different document quality on DBP

Study or Subgroup	Experimental Mean	Experimental SD	Experimental Total	Control Mean	Control SD	Control Total	Weight	Mean Difference IV, Random, 95% CI
**1.20.1 Execise**								
Aileen Wai Kiu Chan 2018	130.88	4.6	49	134.21	6	48	6.2%	-3.33 [-5.46, -1.20]
Lijuan Feng 2018	130.14	13.22	82	140.76	20.97	82	5.6%	-10.62 [-15.99, -5.25]
Xinye Li 2024	135.25	3.23	30	134.36	4.25	30	6.2%	0.89 [-1.02, 2.80]
Zhi-Wei Yan 2022	125.39	11.68	173	127.99	9.69	169	6.2%	-2.60 [-4.87, -0.33]
Zibo Shi 2017	133.42	7.69	36	143.46	11.5	37	5.8%	-10.04 [-14.52, -5.56]
**Subtotal (95% CI)**			**370**			**366**	**29.9%**	**-4.51 [-8.02, -1.00]**
Heterogeneity: Tau² = 13.20; Chi² = 32.36, df = 4 (P < 0.00001); I² = 88%								
Test for overall effect: Z = 2.52 (P = 0.01)								
**1.20.2 Medicine**								
Bo Lin 2021	132.02	5.48	50	139.62	8.85	49	6.1%	-7.60 [-10.51, -4.69]
Chunhua Ma 2018	144.37	17.08	79	148.64	19.46	79	5.5%	-4.27 [-9.98, 1.44]
Huijuan Xie 2014	141.47	10.09	25	152.39	8.71	25	5.6%	-10.92 [-16.15, -5.69]
JEN-CHEN TSAI 2003	126.8	7.4	37	154.6	12.2	39	5.8%	-27.80 [-32.31, -23.29]
Jing Sun 2015	120.28	14.63	136	128.13	14.55	130	6.0%	-7.85 [-11.36, -4.34]
Qiaoying Han 2010	127.9	7.2	30	138.9	8.4	28	5.9%	-11.00 [-15.04, -6.96]
Xiao-Ling Shou 2019	126.68	9.87	98	142.91	6.8	100	6.2%	-16.23 [-18.60, -13.86]
Xiaobin Wang 2019	134.46	9.44	50	122.87	9.28	50	5.9%	11.59 [7.92, 15.26]
Xiaojun Wang 2011	124.69	5.7	30	140.16	20.3	30	5.0%	-15.47 [-23.02, -7.92]
Xiaoling Shou 2018	130.46	9.88	98	138.54	7.97	100	6.2%	-8.08 [-10.58, -5.58]
Yami Cao 2024	127.55	5.82	40	132.53	5.76	40	6.1%	-4.98 [-7.52, -2.44]
Yongcai Zheng 2015	124.48	8.71	49	136.43	9.65	49	6.0%	-11.95 [-15.59, -8.31]
**Subtotal (95% CI)**			**722**			**719**	**70.1%**	**-9.46 [-14.21, -4.71]**
Heterogeneity: Tau² = 66.02; Chi² = 244.49, df = 11 (P < 0.00001); I² = 96%								
Test for overall effect: Z = 3.90 (P < 0.0001)								
**Total (95% CI)**			**1092**			**1085**	**100.0%**	**-8.10 [-11.68, -4.51]**
Heterogeneity: Tau² = 52.87; Chi² = 351.61, df = 16 (P < 0.00001); I² = 95%								
Test for overall effect: Z = 4.42 (P < 0.00001)								
Test for subgroup differences: Chi² = 2.69, df = 1 (P = 0.10), I² = 62.9%								

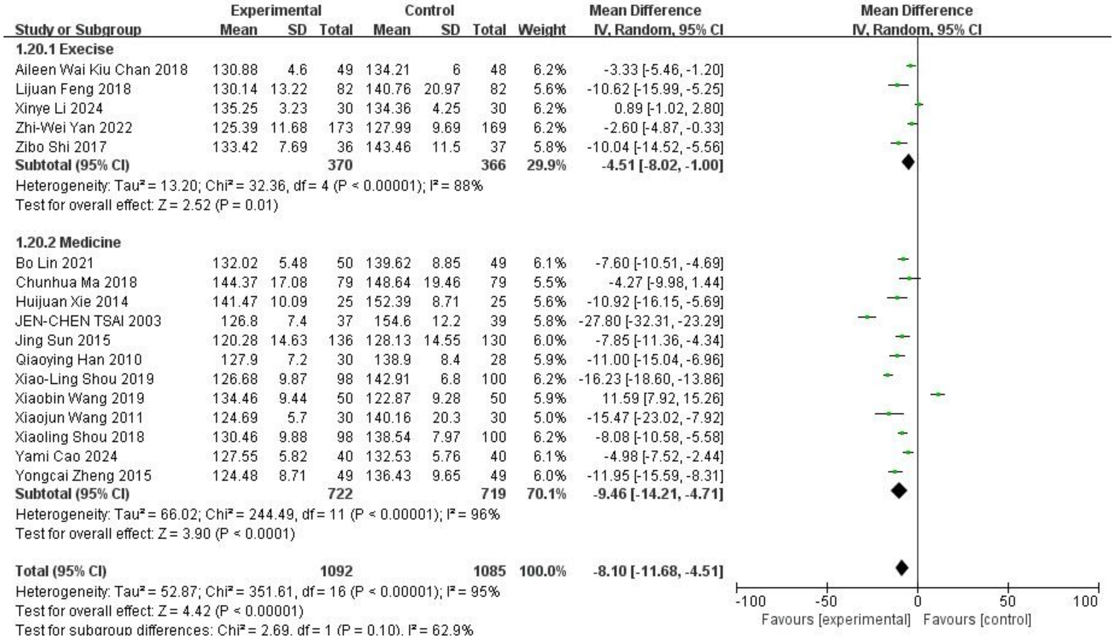

Fig. 16. Forest map of effects of different control measures on SBP

Study or Subgroup	Experimental Mean	Experimental SD	Experimental Total	Control Mean	Control SD	Control Total	Weight	Mean Difference IV, Random, 95% CI
**1.21.1 Execise**								
Aileen Wai Kiu Chan 2018	76.78	9.61	82	83.82	10.19	82	5.8%	-7.04 [-10.07, -4.01]
Lijuan Feng 2018	78.86	7.32	36	81.05	7.33	37	5.6%	-2.19 [-5.55, 1.17]
Xinye Li 2024	80.47	6.98	173	81.94	7.67	169	6.6%	-1.47 [-3.03, 0.09]
Zhi-Wei Yan 2022	79.39	5.25	49	86	5.04	47	6.4%	-6.61 [-8.67, -4.55]
Zibo Shi 2017	81.58	4.31	30	89.48	3.53	30	6.4%	-7.90 [-9.89, -5.91]
**Subtotal (95% CI)**			**370**			**365**	**30.8%**	**-5.05 [-7.98, -2.12]**
Heterogeneity: Tau² = 9.63; Chi² = 34.20, df = 4 (P < 0.00001); I² = 88%								
Test for overall effect: Z = 3.38 (P = 0.0007)								
**1.21.2 Medicine**								
Bo Lin 2021	76.43	6.68	50	81.1	4.31	49	6.3%	-4.67 [-6.88, -2.46]
Chunhua Ma 2018	84.53	8.91	79	87.6	7.78	79	6.1%	-3.07 [-5.68, -0.46]
Huijuan Xie 2014	83.59	9.67	25	98.23	7.43	25	4.7%	-14.64 [-19.42, -9.86]
JEN-CHEN TSAI 2003	78.6	6	37	89.6	7.8	39	5.7%	-11.00 [-14.12, -7.88]
Jing Sun 2015	75.31	14.53	136	79.58	12.44	130	5.7%	-4.27 [-7.52, -1.02]
Qiaoying Han 2010	83.4	6.7	30	87.2	7.1	28	5.5%	-3.80 [-7.36, -0.24]
Xiao-Ling Shou 2019	76.28	7.79	98	83.29	7.8	100	6.3%	-7.01 [-9.18, -4.84]
Xiaobin Wang 2019	76.24	10.23	50	68.44	8.46	50	5.4%	7.80 [4.12, 11.48]
Xiaojun Wang 2011	77.34	5.92	30	84.38	10.98	30	4.9%	-7.04 [-11.50, -2.58]
Xiaoling Shou 2018	82.29	7.82	98	87.2	9.77	100	6.2%	-4.91 [-7.37, -2.45]
Yami Cao 2024	78.63	4.5	40	84.61	4.48	40	6.4%	-5.98 [-7.95, -4.01]
Yongcai Zheng 2015	75.32	3.84	49	84.73	7.84	49	6.2%	-9.41 [-11.85, -6.97]
**Subtotal (95% CI)**			**722**			**719**	**69.2%**	**-5.61 [-7.98, -3.24]**
Heterogeneity: Tau² = 15.06; Chi² = 93.88, df = 11 (P < 0.00001); I² = 88%								
Test for overall effect: Z = 4.63 (P < 0.00001)								
**Total (95% CI)**			**1092**			**1084**	**100.0%**	**-5.43 [-7.22, -3.64]**
Heterogeneity: Tau² = 12.02; Chi² = 130.68, df = 16 (P < 0.00001); I² = 88%								
Test for overall effect: Z = 5.94 (P < 0.00001)								
Test for subgroup differences: Chi² = 0.08, df = 1 (P = 0.77), I² = 0%								

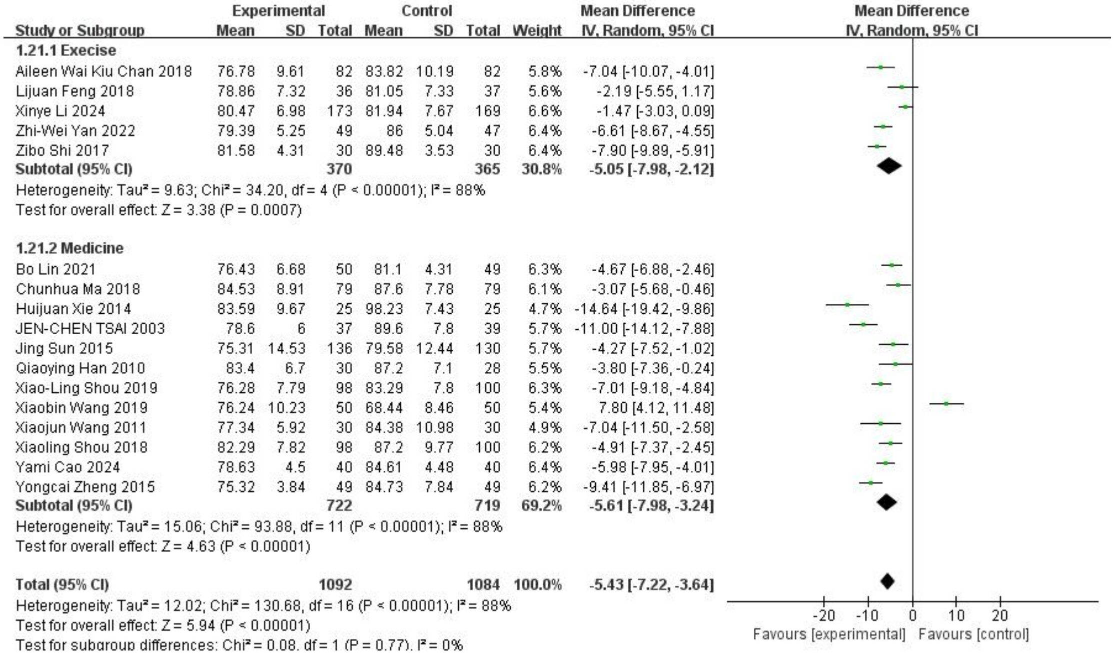

Fig. 17. Forest map of effects of different control measures on DBP
